# Changes in atmospheric circulation and evapotranspiration are reducing rainfall in the Brazilian Cerrado

**DOI:** 10.1038/s41598-023-38174-x

**Published:** 2023-07-11

**Authors:** G. S. Hofmann, R. C. Silva, E. J. Weber, A. A. Barbosa, L. F. B. Oliveira, R. J. V. Alves, H. Hasenack, V. Schossler, F. E. Aquino, M. F. Cardoso

**Affiliations:** 1 Programa de Pós-Graduação em Geografia, Universidade Federal do Rio Grande do Sul, Porto Alegre, RS Brazil; 2 Laboratório de Geoprocessamento, Centro de Ecologia, Universidade Federal do Rio Grande do Sul, Porto Alegre, RS Brazil; 3 Departamento Interdisciplinar, Universidade Federal do Rio Grande do Sul, Tramandaí, RS Brazil; 4 Programa Pós-Graduação em Sensoriamento Remoto, Universidade Federal do Rio Grande do Sul, Porto Alegre, RS Brazil; 5 Earth System Sciences, National Institute for Space Research, Instituto Nacional de Pesquisas Espaciais, São José Dos Campos, SP Brazil; 6 Departamento de Vertebrados, Museu Nacional, Universidade Federal do Rio de Janeiro, Rio de Janeiro, RJ Brazil; 7 Departamento de Botânica, Museu Nacional, Universidade Federal do Rio de Janeiro, Rio de Janeiro, RJ Brazil; 8 Programa de Pós-Graduação em Agronegócios, Centro de Pesquisas em Agronegócios, Universidade Federal do Rio Grande do Sul, Porto Alegre, RS Brazil

**Keywords:** Climate sciences, Environmental sciences

## Abstract

Here we analyze the trends of rainfall and the frequency of rainy days over the Brazilian Cerrado between 1960 and 2021 in four distinct periods according to the seasonal patterns over the region. We also evaluated trends in evapotranspiration, atmospheric pressure, winds, and atmospheric humidity over the Cerrado to elucidate the possible reasons for the detected trends. We recorded a significant reduction in rainfall and frequency of rainy days in the northern and central Cerrado regions for all periods except at the beginning of the dry season. The most pronounced negative trends were recorded during the dry season and the beginning of the wet season, where we recorded reductions of up to 50% in total rainfall and the number of rainy days. These findings are associated with the intensification of the South Atlantic Subtropical Anticyclone, which has been shifting atmospheric circulation and raising regional subsidence. Moreover, during the dry season and the beginning of the wet season, there was a reduction in regional evapotranspiration, which also potentially contributed to the rainfall reduction. Our results suggest an expansion and intensification of the dry season in the region, potentially bringing broad environmental and social impacts that transcend the Cerrado boundaries.

## Introduction

**Figure 1:**
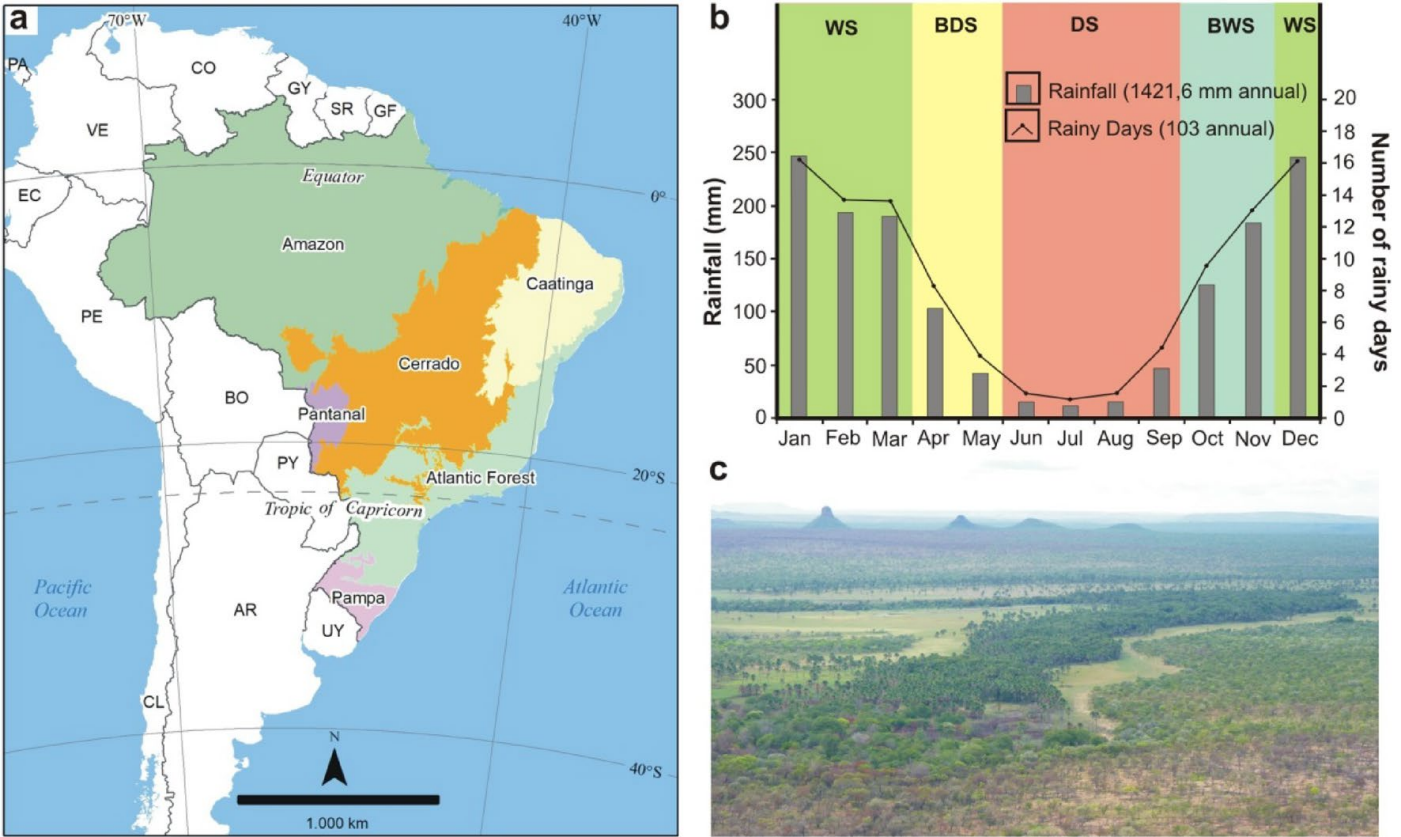
Location of the Brazilian Cerrado and its annual rainfall cycle. (**a**) Official limits of the Cerrado and other Brazilian ecoregions. (**b**) Monthly means of total rainfall (gray bars) and the frequency of rainy days (black line) in Cerrado between 1960 and 1990 (here considered as period reference), where the values were calculated by the average of all 70 pluviometric stations used in this study*.* The acronyms WS, BDS, DS, and BWS represent the Wet Season, Beginning of Dry Season, Dry Season, and Beginning of Wet Season, respectively. (**c**) Panoramic view of the Cerrado vegetation during the dry season, near Terra Ronca State Park (state of Goiás). The map in the upper level was produced using ArcGIS (https://www.arcgis.com).

Brazil has been a main international focus point concerning biological conservation and global climate change, primarily due to the size and high diversity of its forests. However, while most of the attention of scientists and decision-makers remains focused on the Amazon and the Atlantic Forest, the other non-forest ecoregions in Brazil are undergoing an accelerated destruction process due to the expansion of the agricultural frontier^1^. This is the case of the Brazilian Cerrado (also called Neotropical Savanna, and hereinafter referred to as Cerrado), a global biodiversity hotspot that extends over 2 million km^2^ and has already lost 50% of its native vegetation cover^2,3^ (Fig. 1a). The Cerrado vegetation is a mosaic of native grasslands, savannas, and forests, and their conversion into pasture or cropland can be associated with a pronounced climate change at local and regional scales^4^. These land use and cover changes are linked to shifts in the local energy budget, especially in the latent heat flux due to the significant reduction in evapotranspiration^5,6^, resulting in higher temperatures and lower relative humidity near the ground^4^. In addition to air temperature and humidity shifts, theoretical simulations evaluating the climatic effects of savanna suppression have predicted a large reduction in precipitation due to both the reduced moisture flux from the land surface as well as weakened moisture convergence into the savanna's core areas^7,8^. According to these projections, the reduction in rainfall could exceed 300 mm/year, representing more than 20% of the annual amount.

Recently, other studies already found trends of reduced rainfall for the Cerrado regions corroborating these theoretical forecasts^9–11^. This reduction in rainfall over the Cerrado is probably not only associated with land use and land cover changes. Since the late 1950s, several studies have found significant changes in atmospheric circulation over the tropical region, especially by an intensification in the Hadley and Walker cells^12–15^. There is consistent evidence of a widening of the Hadley cell's poleward edge during the austral summer on the South Atlantic Ocean due to increasing greenhouse gases and decreasing stratospheric ozone concentrations in the Southern Hemisphere^16^. The South Atlantic Subtropical Anticyclone (SASA) is one of three anticyclones that form in Hadley cell's poleward edge in the oceans of the Southern Hemisphere and controls the climatic seasonality in all Brazilian tropical regions. Variations in the location and intensity of SASA deeply affect the Brazilian weather through changes in atmospheric pressure, air subsidence intensity, winds, and humidity flux that reach the continental areas^17^. Therefore, these alterations in the Hadley cell can lead to deep impacts on the hydrological cycle and regional energy budget in the continental areas of South America. For instance, the increasing tropospheric and surface dryness over the Brazilian tropical region is projected by models considering Hadley cells circulation in different CO_2_ warming scenarios^18^. So far, the consequences of strengthening the SASA next to the Brazilian coast have yet to be fully known, as well as the likely changes in atmospheric circulation and hydrological cycle over the adjacent tropical continental areas^17,19^.

Like other areas of tropical savannas, the climate in the Cerrado is characterized by high temperatures throughout the year, with wet and dry seasons occurring in the austral summer and winter, respectively^20^. The annual rainfall ranges from 1800 mm in the transition zones with the Amazon rainforest to 1000 mm in the areas bordering the Caatinga (a semi-arid ecoregion in northeastern Brazil)^21,22^. The weather stability of the dry season in the Cerrado is a consequence of the subsidence generated by the SASA that inhibits the moisture convection and the vertical development of clouds^23^. During this period, the prevailing winds blow from east to west, and the humidity transport from the Atlantic Ocean and Amazon forest is minimal compared to the summer^24^. The summer wet season is associated with the South American Monsoon System (SAMS), a period in which both the Intertropical Convergence Zone (ITCZ) and the South Atlantic Convergence Zone (SACZ) occur in the region^25^. The wet season begins between late September and the beginning of November, when the Southern Hemisphere becomes progressively warmer due to the greater incidence of solar radiation, weakening and moving away the SASA from the South American continent^17^. The progressive heating of the continent reverses the direction of 850-hPa zonal winds, reduces the atmospheric pressure over South America, and intensifies the moisture flow from the Atlantic Ocean and the Amazon forest into the Cerrado^26^. So, the low atmospheric pressure and high humidity over the Cerrado favored the deep convection and occurrence of intense rainfall events. From the end of March, convection gradually migrates to the equatorial region, and moisture transport from the Amazon weakens, ending the wet season in the Cerrado^24^.

In this study, our main objective is to further investigate trends in the amount and frequency of rainfall over the Cerrado between 1960 and 2021, and relate them to other environmental variables. For that, we also evaluated changes in evapotranspiration, atmospheric pressure, winds, and atmospheric humidity over the Cerrado to elucidate possible shifts in atmospheric circulation and the SAMS in the same period. Therefore, our analyses cover the period from the first years of Hadley cell intensification/expansion records to the present, and date back to the period before agricultural expansion in the region. We performed all data analyses in four distinct periods according to the rainfall distribution patterns over Brazilian tropical areas throughout the year^23,24^. These periods are the wet season (WS; formed by December, January, February, and March), the beginning of the dry season (BDS; April and May), the dry season (DS; June, July, August, and September), and the beginning of the wet season (BWS; October and November) (Fig. 1b).

## Results

### Rainfall reduction over Cerrado

**Figure 2:**
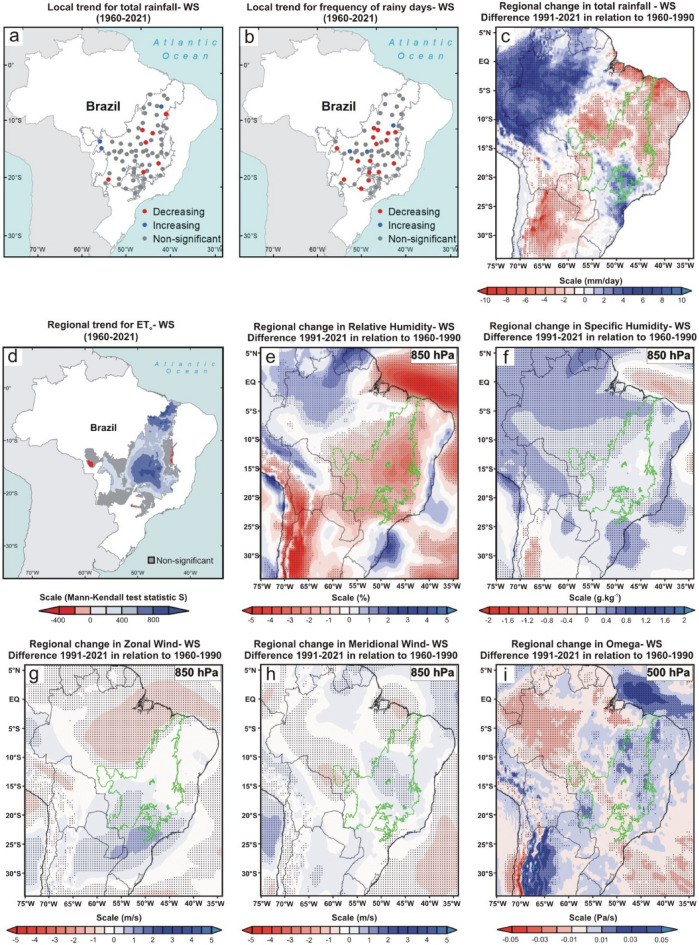
Climate changes over the last six decades during the wet season (WS; December, January, February, and March) of the Brazilian Cerrado. (**a**, **b**, and **d**) Mann–Kendall test over 1960–2021 for total rainfall (mm), frequency of rainy days, and reference evapotranspiration (ET_o_; mm), respectively. The blue, red, and gray colors represent the areas with a significant increasing trend (*p* < 0.05), significant decreasing trend (*p* < 0.05), and non-significant trend, respectively. The blue and red color gradient for reference evapotranspiration analyses represents Mann–Kendall’s S-statistics results for all pixels with a significant trend. (**c**, **e**–**i**) Regional changes detected by ERA5 reanalysis for total rainfall (mm/day), relative humidity (%) at 850-hPa, specific humidity (g kg^-1^) at 850-hPa, horizontal speed of zonal wind (m/s), horizontal speed of meridional wind (m/s) at 850-hPa, and vertical velocity (omega) (Pa/s) at 500-hPa, respectively. In all reanalysis, the dotted areas demonstrated a significant difference (*p* < 0.05) between the climate normals 1991–2021 and 1960–1990. The gray and green polygons show the official limits of the Brazilian Cerrado in the Mann–Kendall tests and reanalysis, respectively. Maps showing local scale trends were generated using ArcGIS (https://www.arcgis.com). The ET_o_ results map was generated using Google Earth Engine (https://earthengine.google.com/). The ERA5 reanalysis maps were generated using GrADS-Grid Analysis and Display System (http://opengrads.org).

**Figure 3:**
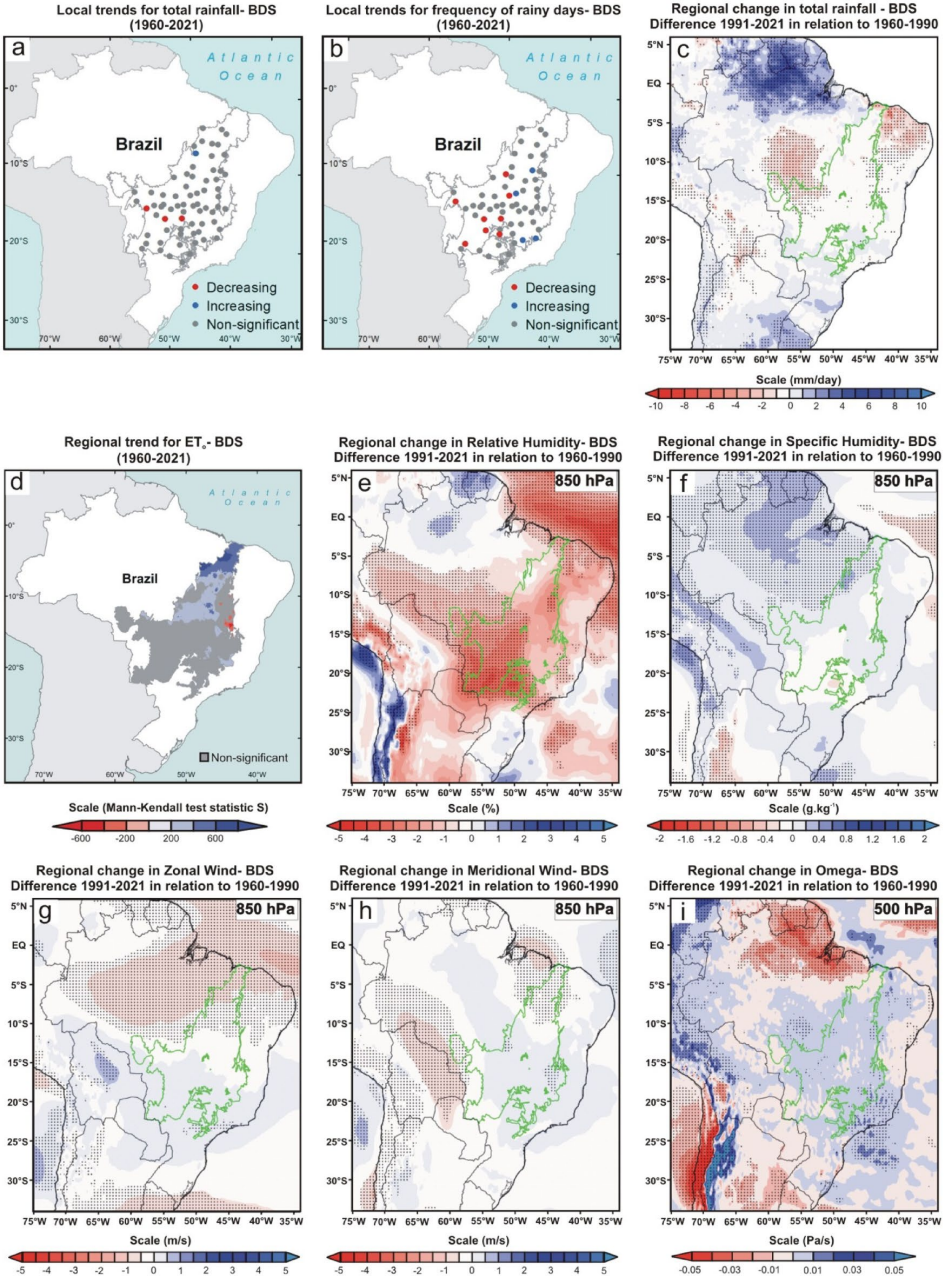
Climate changes over the last six decades during the beginning of the dry season (BDS; April and May) of the Brazilian Cerrado. (**a**, **b**, and **d**) Mann–Kendall test over 1960–2021 for total rainfall (mm), frequency of rainy days, and reference evapotranspiration (ET_o_; mm), respectively. The blue, red, and gray colors represent the areas with a significant increasing trend (*p* < 0.05), significant decreasing trend (*p* < 0.05), and non-significant trend, respectively. The blue and red color gradient for reference evapotranspiration analyses represents Mann–Kendall’s S-statistics results for all pixels with a significant trend. (**c**, **e–i**) Regional changes detected by ERA5 reanalysis for total rainfall (mm/day), relative humidity (%) at 850-hPa, specific humidity (g kg^-1^) at 850-hPa, horizontal speed of zonal wind (m/s), horizontal speed of meridional wind (m/s) at 850-hPa, and vertical velocity (omega) (Pa/s) at 500-hPa, respectively. In all reanalysis, the dotted areas demonstrated a significant difference (*p* < 0.05) between the climate normals 1991–2021 and 1960–1990. The gray and green polygons show the official limits of the Brazilian Cerrado in the Mann–Kendall tests and reanalysis, respectively. Maps showing local scale trends were generated using ArcGIS (https://www.arcgis.com). The ET_o_ results map was generated using Google Earth Engine (https://earthengine.google.com/). The ERA5 reanalysis maps were generated using GrADS-Grid Analysis and Display System (http://opengrads.org).

**Figure 4:**
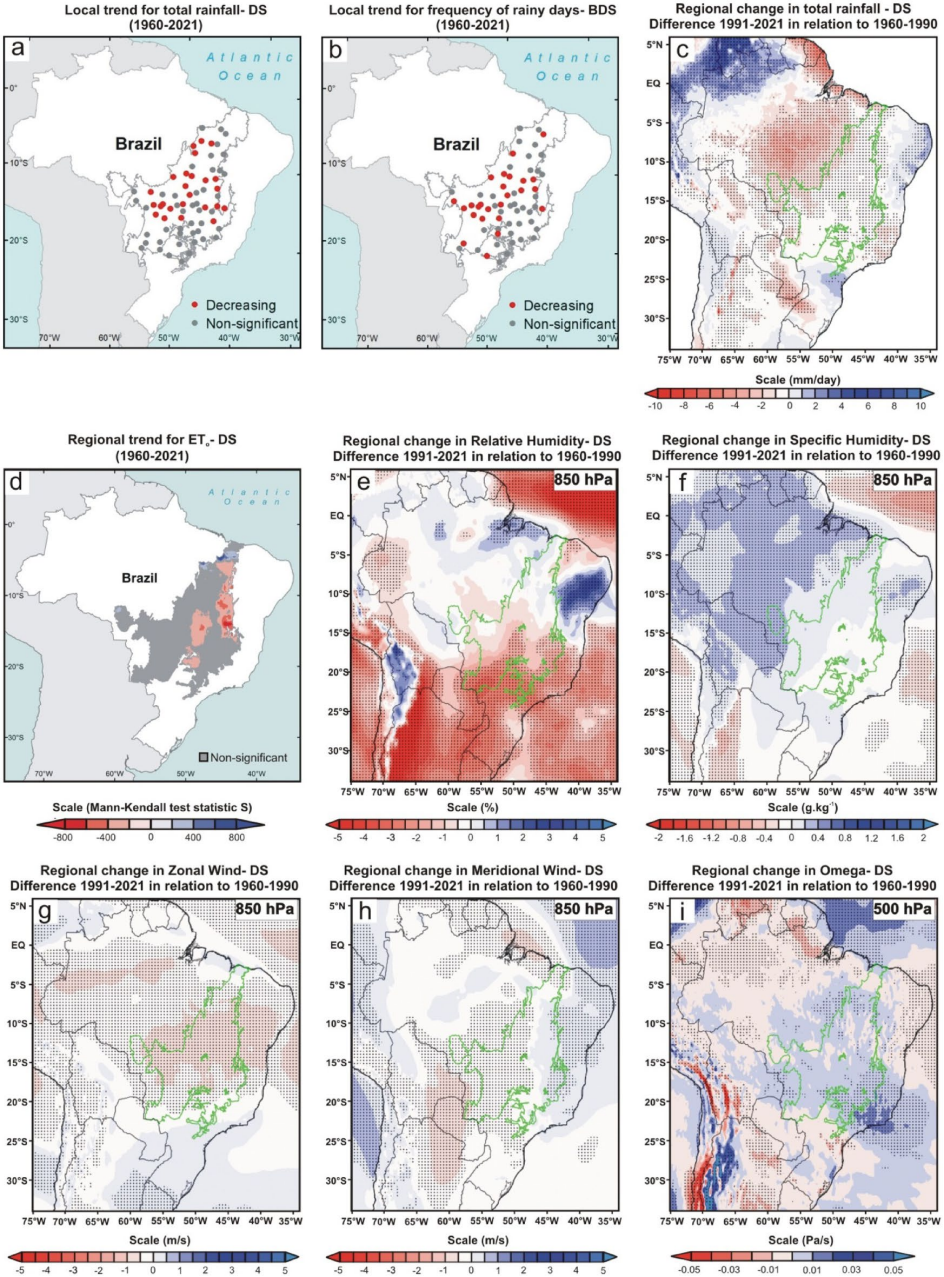
Climate changes over the last six decades during the dry season (DS; June, July, August and September) of the Brazilian Cerrado. (**a**, **b**, and** d**) Mann–Kendall test over 1960–2021 for total rainfall (mm), frequency of rainy days, and reference evapotranspiration (ET_o_; mm), respectively. The blue, red, and gray colors represent the areas with a significant increasing trend (*p* < 0.05), significant decreasing trend (*p* < 0.05), and non-significant trend, respectively. The blue and red color gradient for reference evapotranspiration analyses represents Mann–Kendall’s S-statistics results for all pixels with a significant trend. (**c**, **e–i**) Regional changes detected by ERA5 reanalysis for total rainfall (mm/day), relative humidity (%) at 850-hPa, specific humidity (g kg^-1^) at 850-hPa, horizontal speed of zonal wind (m/s), horizontal speed of meridional wind (m/s) at 850-hPa, and vertical velocity (omega) (Pa/s) at 500-hPa, respectively. In all reanalysis, the dotted areas demonstrated a significant difference (*p* < 0.05) between the climate normals 1991–2021 and 1960–1990. The gray and green polygons show the official limits of the Brazilian Cerrado in the Mann–Kendall tests and reanalysis, respectively. Maps showing local scale trends were generated using ArcGIS (https://www.arcgis.com). The ET_o_ results map was generated using Google Earth Engine (https://earthengine.google.com/). The ERA5 reanalysis maps were generated using GrADS-Grid Analysis and Display System (http://opengrads.org).

**Figure 5:**
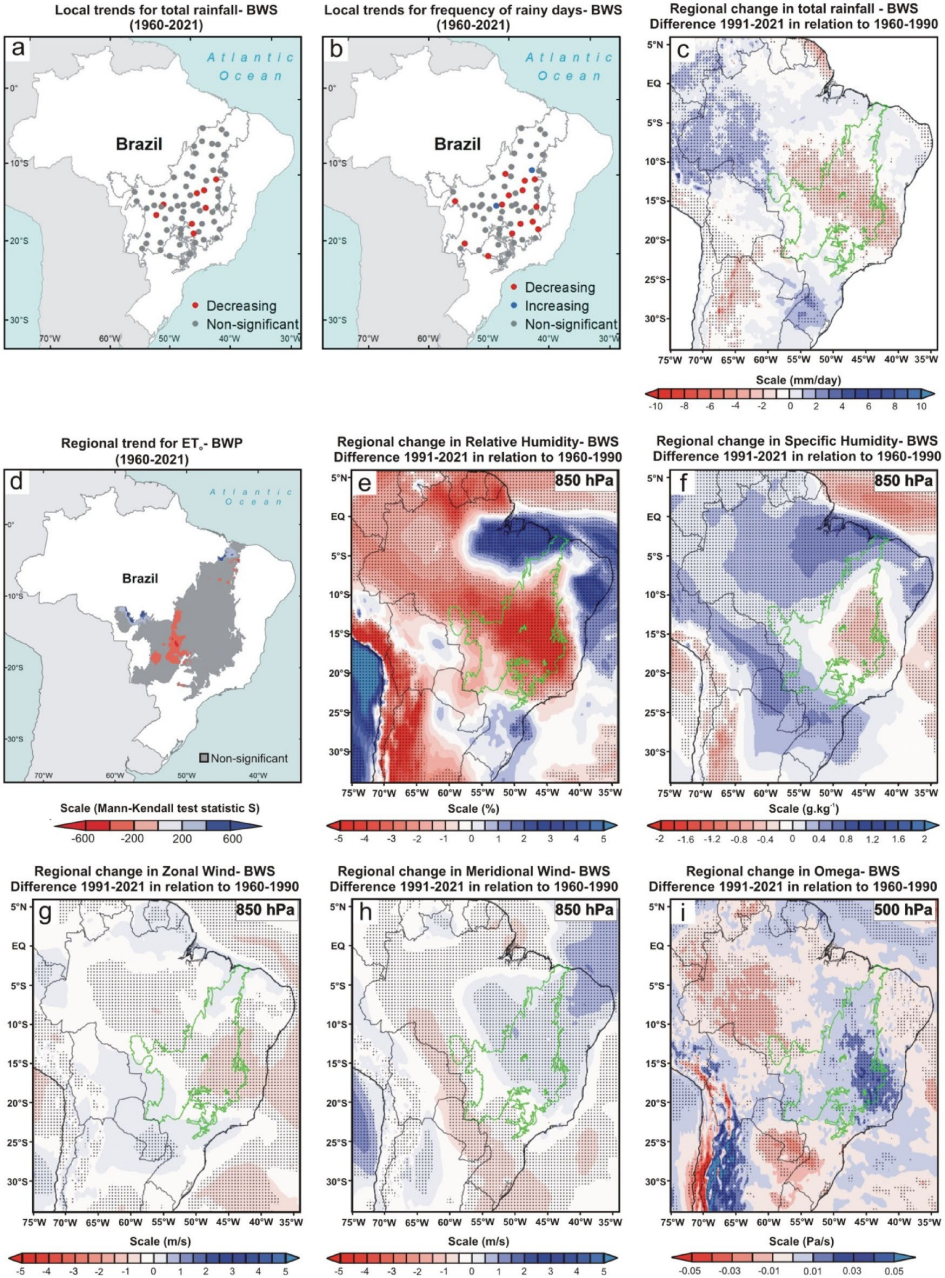
Climate changes over the last six decades during the beginning of the wet season (BWS; October and November) of the Brazilian Cerrado. (**a**, **b**, and **d**) Mann–Kendall test over 1960–2021 for total rainfall (mm), frequency of rainy days, and reference evapotranspiration (ET_o_; mm), respectively. The blue, red, and gray colors represent the areas with a significant increasing trend (*p* < 0.05), significant decreasing trend (*p* < 0.05), and non-significant trend, respectively. The blue and red color gradient for reference evapotranspiration analyses represents Mann–Kendall’s S-statistics results for all pixels with a significant trend. (**c**, **e**–**i**) Regional changes detected by ERA5 reanalysis for total rainfall (mm/day), relative humidity (%) at 850-hPa, specific humidity (g.kg^-1^) at 850-hPa, horizontal speed of zonal wind (m/s), horizontal speed of meridional wind (m/s) at 850-hPa, and vertical velocity (omega) (Pa/s) at 500-hPa, respectively. In all reanalysis, the dotted areas demonstrated a significant difference (*p* < 0.05) between the climate normals 1991–2021 and 1960–1990. The gray and green polygons show the official limits of the Brazilian Cerrado in the Mann–Kendall tests and reanalysis, respectively. Maps showing local scale trends were generated using ArcGIS (https://www.arcgis.com). The ET_o_ results map was generated using Google Earth Engine (https://earthengine.google.com/). The ERA5 reanalysis maps were generated using GrADS-Grid Analysis and Display System (http://opengrads.org).

**Figure 6:**
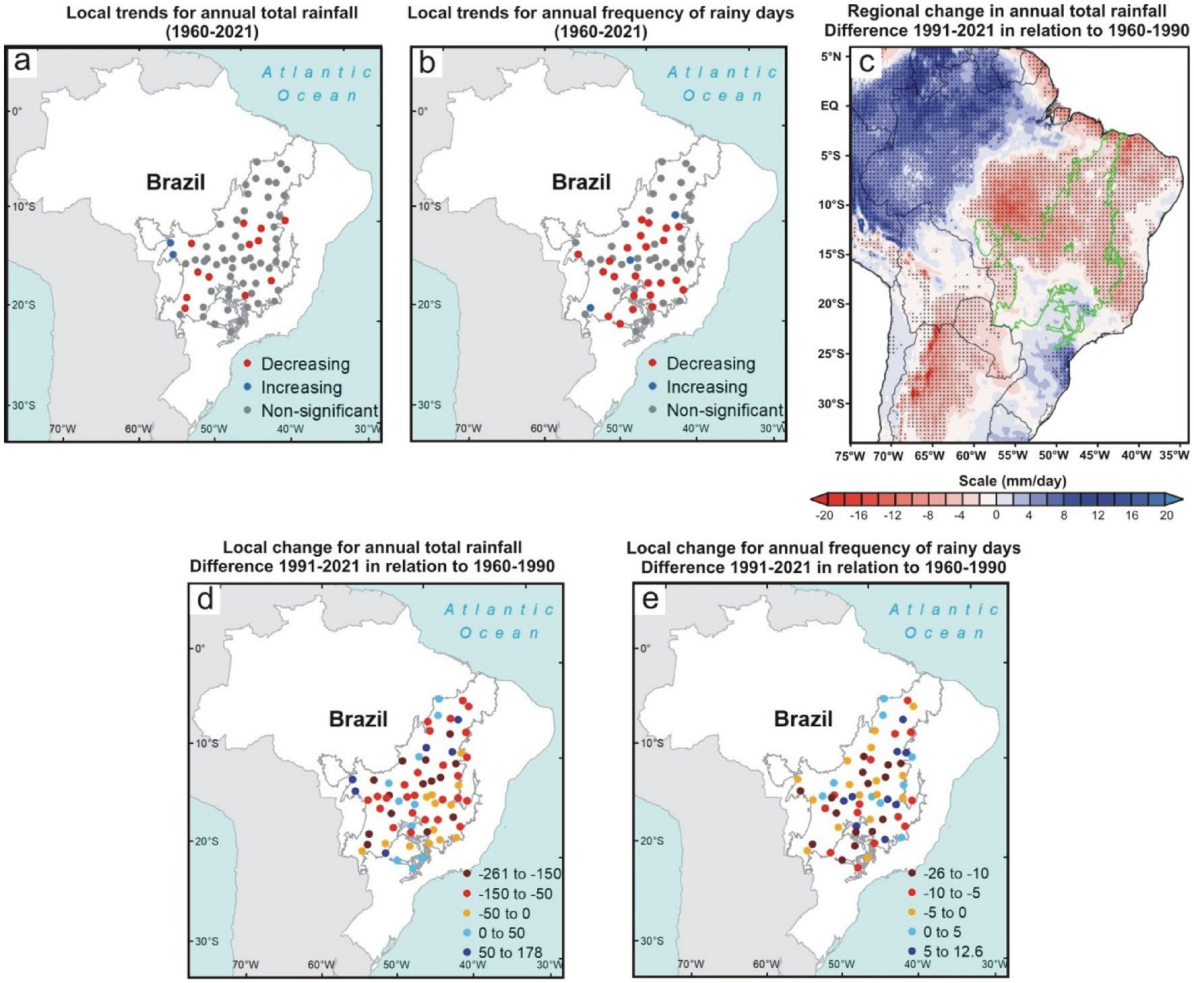
Change in annual total rainfall and annual frequency of rainy days in the Brazilian Cerrado between 1960 and 2021. (**a**, **b**) Mann–Kendall test over 1960–2021 for annual total rainfall (mm) and frequency of rainy days, respectively. The blue, red, and gray colors represent the areas with a significant increasing trend (*p* < 0.05), significant decreasing trend (*p* < 0.05), and non-significant trend, respectively. (**c**) Regional changes detected by ERA5 reanalysis for annual total rainfall (mm/day). The dotted areas demonstrated a significant difference (*p* < 0.05) between the climate normals 2021–1991 and 1960–1990. (**d** and **e**) Local changes in annual total rainfall and annual frequency of rainy days by comparing the climate normal 1991–2021 to the climate normal 1960–1990, respectively. Maps showing local scale trends were generated using ArcGIS (https://www.arcgis.com). The ERA5 reanalysis map were generated using GrADS-Grid Analysis and Display System (http://opengrads.org).

As a starting point, we define the 1960–1990 climate normal as a reference value to estimate the changes in rainfall in the Cerrado. Our analyses were based on data from direct observation in 70 pluviometric stations spread across all Cerrado sub-regions (i.e., local scale) and from a combination of model data with observations generated by the ERA5 global reanalysis (regional scale) (Methods). At the local scale, the Mann–Kendall test showed that most localities have a downward trend for both total rainfall and the frequency of rainy days (Figs. 2, 3, 4, 5, 6a and b). In all analyzed periods, we found more significant cases in the frequency of rainy days than total rainfall. A similar result has already been observed in a study limited to northern Cerrado and considering a shorter sample period^10^. However, this difference may also be partially associated with the high variance of rainfall data in Cerrado, impairing model fitting, even non-parametric ones, such as the Mann–Kendall test. The high variance of precipitation data is a characteristic that can also be observed in the other tropical ecoregions of Brazil^23,27,28^. In our study, for example, the last two years of the series (2020 and 2021) were exceptionally wet due to high-intensity precipitation events (above 100 mm/day).

Results from reanalysis data corroborated the local trends showing a significant reduction of total rainfall in the central portion and no significant changes in the southern Cerrado (Fig. 2, 3, 4, 5, 6c). However, only the reanalysis showed a significant reduction in the northern portion. Again, we attribute this disagreement to the difficulty of fitting the models to the high variance in the observed total rainfall because the negative trends far outweigh the positive ones in the northern Cerrado, even if the fitting are not statistically significant. These non-significant reduction trends of total rainfall can be seen by comparing the mean of the last 31 years (1991–2021) to that of the first 31 years (1960–1990) because 54 of the 70 pluviometric stations have a reduction in annual rainfall (Fig. 6d; Supplementary Table S1). From this comparison, considering the mean of the 70 monitoring sites, we calculated that the annual reduction was 64.8 mm in precipitation and of 5.2 days in the number of rainy days, a reduction similar to that found in previous studies^11^. Nonetheless, both values represent overall estimates for the entire Cerrado and, in many sites, these reductions are greater than twice these means (Fig. 6d–e; Supplementary Tables S1 and S2).

The DS is when the trend towards reduced precipitation and frequency of rainy days is more consistent, occurring practically in the entire Cerrado (i.e., 67 of the 70 monitoring sites; Fig. 4a–c; Supplementary Fig. S1c). This period was also the one in which the reductions in rainfall amount and frequency of rainy days were proportionally higher compared to the 1960–1990 climate normal, with dozens of monitoring sites having reductions greater than 30% in both variables (Supplementary Tables S1 and S2). As shown in Fig. 4c, the significant reduction in precipitation during the DS goes beyond the Cerrado boundaries, extending to neighboring ecoregions such as the Pantanal (west edge), south and east of the Amazon (northwest edge), part of the Atlantic Forest (southeast edge) and a narrow portion of Caatinga (northeast edge). Other studies conducted in some of these ecoregions have confirmed this outcome^10,27,29,30^. We also detected a significant reduction in rainfall during the BWS in the central Cerrado, extending to the southern Amazon and the center of the Atlantic Forest (Fig. 5a–c). This change is important because the expansion of the DS is one of the main predictions of theoretical simulations evaluating the climatic effects of savanna supression^7^ and has also been observed by other studies in the south and east of the Amazon^10,14^. During the WS, rainfall was significantly reduced in central and northern Cerrado (Fig. 2a–c), encompassing the southeastern Amazon, Pantanal, and the entire northeastern region of Brazil. The BDS is the period when the changes were less noticeable, with significant reduction trends of the total rainfall occurring only in the western edge of the Cerrado (Fig. 3a–c), extending to the south and east of the Amazon^30^. In summary, we found a broad tendency toward an annual reduction in total rainfall and in the frequency of rainy days in almost all Cerrado (Fig. 6a–c). Moreover, we observed this same pattern for large areas of other Brazilian tropical ecoregions such as Pantanal, Caatinga, Atlantic Forest, and southern Amazon (Fig. 6c).

### Changes in evapotranspiration, air humidity, and atmospheric circulation

From the TerraClimate dataset, we again used the Mann-Kendell test to analyze the trend in reference evapotranspiration (ET_o_; based on the Penman-Montieth method) for the entire Cerrado between 1960 and 2021 (Methods). We recorded positive ET_o_ trends in large areas in the Cerrado during the WS and at the BDS (Figs. 2, 3d). This trend is directly associated with the recent warming of the Cerrado, which has been raising the regional evaporative demand, consequently increasing the evapotranspiration rates in periods of high water availability^31,32^. We believe that ET_o_ is a good approximation to evaluate evapotranspiration in our case, give the predominance of grassland and savanna ecosystems and the high availability of water during the summer months in Cerrado, which are factors that support the trends found at the WS and BDS^33^. Additionally, the extensive conversion of native vegetation into agriculture and pasture does not seem to be a factor that can shift the trends in these two periods because some cropping systems in the Cerrado have evapotranspiration rates similar to or greater than natural vegetation between December and May when cultures are at an advanced stage of growth^34^.

On the other hand, we recorded a reduction in evapotranspiration in extensive areas of the Cerrado in the DS and BWS (Figs. 4, 5d). This ET_o_ reduction is not directly associated with land use and land cover changes because its formulation uses a static reference land cover. Instead, the increase in evaporative demand during the DS in Cerrado may have been so high that it possibly exceeded the pre-established physiological capacity for the grass reference surface in the Penman-Montieth equation (i.e., a fixed “bulk” surface resistance of 70 sm^-1^)^33^. Contrary to the assumption in the calculation of ET_o_, where there is no water restriction for grass reference surface, the plants of Cerrado are subject to a severe limitation of water supply during the DS and BWS^32^, which probably potentiates the reduction of real evapotranspiration. Even if we could not explicitly consider land use and land cover changes in our study, this process results in the evapotranspiration decrease in Cerrado because, contrary to what happens in agricultural areas, the deep root system of part of the Cerrado native vegetation allows high evapotranspiration rates in DS and BWS^5,6^. For all these reasons, we conclude that the trend of reduction in evapotranspiration during these two periods is a reality and that it can be aggravated if the suppression of Cerrado native vegetation is maintained in the coming years.

We recorded significant reductions in relative humidity in the 850-hPa layer (i.e., ~ 1.5 km above sea level) between the two climate normals in all periods analyzed (Figs. 2, 3, 4, 5e). However, the same approach found no significant changes in specific humidity in the same atmospheric layer (Figs. 2, 3, 4, 5f). This apparent contradiction can be explained by the regional warming^4^ and the intensification of the SASA over the past three decades, leading to increased subsidence over the Cerrado. The compression of the air mass during the subsidence process leads to adiabatic heating, raising the vapor pressure deficit and reducing the relative humidity. Additionally, the air subsidence prevents deep convection, explaining why we do not observe a significant increase in specific humidity during the wet season and at the beginning of the dry season, periods in which we recorded rises in surface evapotranspiration. Here we demonstrate this enhancement of the SASA by the increase in the horizontal speed of air moving toward the west during the dry season and the beginning of the wet season (i.e., negative values of the zonal wind; Figs. 4, 5g) and increase in the vertical velocity of wind (omega) in all periods (Figs. 2, 3, 4, 5i). We highlight the increase in vertical velocity of the wind at 500-hPa (i.e., ~ 5.5 km above sea level), but we also revealed omega changes in other layers of the troposphere in four meridians that cover almost the entire longitudinal extension of the Cerrado (Supplementary Figs. S3–S6).

**Figure 7:**
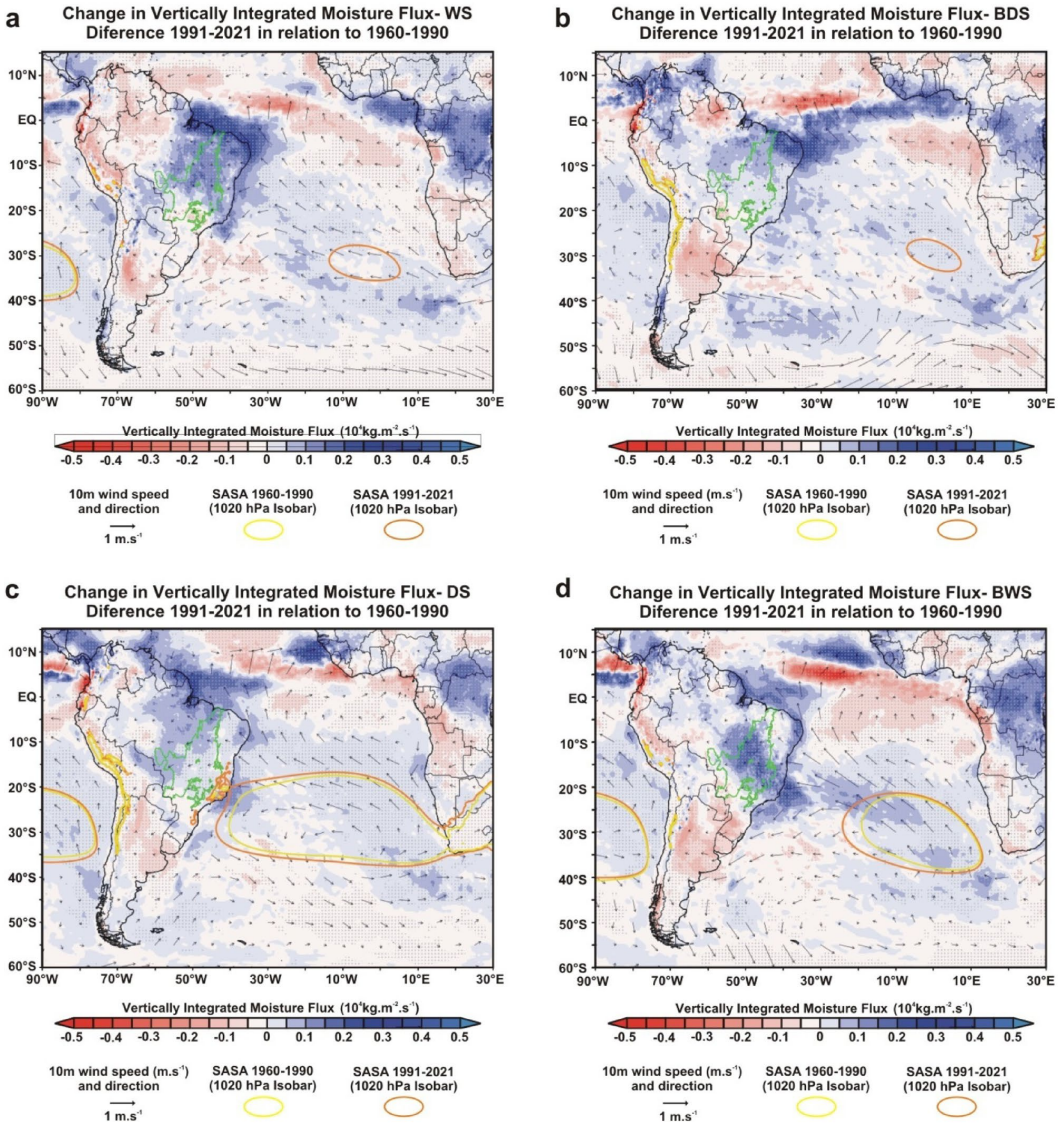
Changes in the Vertically Integrated Moisture Flux and the intensity and direction of 10 m Wind between 1960 and 2021 in the South America/South Atlantic domain. (**a**–**d**) Changes in Vertically Integrated Moisture Flux and 10 m Winds recorded by ERA5 reanalysis in the Wet Season (WS), Beginning of Dry Season (BDS), Dry Season (DS), and Beginning of Wet Season (BWS), respectively. The dotted areas demonstrated a significant difference (*p* < 0.05) in the Vertically Integrated Moisture Flux between 1991–2021 and 1960–1990 climate normals. Black vectors show the changes in direction and intensity of 10 m Wind. The green polygon shows the official limits of the Brazilian Cerrado. The yellow and orange ellipses represent the 1020 hPa isobar and show the South Atlantic Subtropical Anticyclone (SASA) position/extension in 1960–1990 and 1991–2021, respectively. Maps in the upper level were produced using GrADS-Grid Analysis and Display System (http://opengrads.org).

Finally, to demonstrate the role of SASA intensification/expansion in reducing rainfall in the Cerrado, we performed two additional analyses where we expanded the spatial scale of the study to include the entire South America/South Atlantic domain. Again, we used the same approach as in the previous analyses and compared the 1991–2021 and 1960–1990 climate normals for all four periods. First, we analyzed the Mean Sea Level Pressure to demonstrate the increase in SASA intensity (Supplementary Fig. S7). Second, we analyzed and overlapped the Vertically Integrated Moisture Flux and the intensity and direction of 10 m Wind in the same figure (Fig. 7). In both analyses, we used the 1020 hPa isobar to show the SASA position/extension, the criterion used by other recent studies^35,36^. The results of these two analyses confirm the expansion of the SASA by the increase of the area occupied by the 1020 hPa isobar during the DS and BWS, in addition to its appearance in the WS and BDS in the period 1991–2021 (note that it did not occur in the South Atlantic between 1960 and 1960; Fig. 7). The intensification of the anticyclone can also be seen by the increase in Sea Level Pressure under most of the South Atlantic and South America (Supplementary Fig. S7) and by the length of the 10 m Wind vectors flowing from the SASA towards the Brazilian coast (Fig. 7). As a result of the SASA intensification, there is an increase in moisture divergence under the tropical region of Brazil that includes most of the Cerrado in all periods analyzed. In other words, the positive values of the Vertically Integrated Moisture Flux indicate that the moisture is spreading out of the Cerrado and converging to other regions of South America (Fig. 7). In this sense, we highlight some changes in meridional winds at 850-hPa that can indicate the fate of this moisture. During the BWS, we found an increase in the horizontal speed of air moving northwards over the central and northern portions of the Cerrado (Fig. 3h). At the same time, we registered a possible intensification of the South American Low-Level Jet in the extreme west edge of the Cerrado, represented by a narrow strip of air flux moving southwards at 850-hPa. In addition to the increase in meridional wind speed, the South American Low-Level Jet also has a significant humidity rise that extends to southern Brazil (Fig. 3f), probably associated with increased rainfall in this region (Fig. 3c), including the occurrence of extreme events of precipitation such as explosive cyclones^37^. Regarding the Cerrado, the increase in the horizontal speed of air moving toward the north during the BWS and WS in its central portion may also be associated with reduced rainfall, as the primary moisture sources for the region in these periods come from the northern sector, especially from the Amazon^26,38^.

## Discussion

Here we presented analyses that confirmed the observations and predictions of empirical and theoretical studies, respectively, and demonstrated rainfall reduction in the Cerrado over the last six decades on local and regional scales^7–10^. Additionally, we found significant changes in evapotranspiration, air humidity, and atmospheric circulation that help to understand the rainfall reduction in each period of the year. By a visual comparison of the results shown in Figs. 2, 3, 4, 5, one can perceive a high spatial overlap between the reduction of total rainfall and relative humidity at 850-hPa with the increase in the vertical velocity of wind at 500-hPa, suggesting that this is a major factor driving changes in the hydrological cycle of the Cerrado because it hampers the vertical development of clouds and water condensation. In this sense, the role played by the omega intensification is more evident during WS, when we found a pronounced reduction and relative humidity at 850-hPA, even with a significant tendency to increase regional evapotranspiration in Cerrado. As explained earlier, this reduction in relative humidity at 850-hPA occurs due to adiabatic heating during the air subsidence process. Our results also showed an intensification of the SASA has led to broad changes in the atmospheric circulation in the South America/South Atlantic domain and transferred the moisture from the tropical regions of Brazil to the other areas of South America, such as the northwest Amazon and the La Plata Basin. We reiterate that this climate change transcends the Cerrado boundaries and reduces rainfall in other Brazilian ecoregions such as Caatinga, Pantanal, and southern Amazon ^14,15,27,28^. Regarding Cerrado, the evapotranspiration reduction during the DS and the BWS acts in synergy with omega increase, causing an intensification and expansion of the dry period in the Cerrado^30^. The combination of effects from evapotranspiration reduction and moisture transference linked to changes in atmospheric circulation are also discussed and recognized as important by a recent study that evaluated the rainfall reduction during the dry season in the southwest of the Amazon in the last 40 years^39^. Although we have not been able to quantify the role of land cover change in this reduction in evapotranspiration, recent studies formally demonstrate these using different methodologies^5,6,32^. Therefore, a process similar to what occurs in the Amazon and which is notoriously related to the reduction of rainfall in this region^40^. Although probable, the effects of massive Amazon deforestation on rainfall in the Cerrado are still unknown. With the reduction of rainfall, there is a trend toward water deficits for most of the Cerrado^32^, which will result in impacts and challenges for the region's future.

The environmental and social impacts of the reduction in the frequency of rainy days and total rainfall are complex and likely to transcend the Cerrado and reach other regions. For instance, this ecoregion contains the headwaters of some of the main Brazilian watersheds (e.g., Parnaíba, Paraná, Paraguay, Tocantins–Araguaia, São Francisco, and rivers tributary to the southern Amazon river)^41^. In this context, the Pantanal likely will be the region most affected by the reduction in rainfall because its floods cycle is directly associated with the flow of rivers from the Cerrado plateaus to the interior of this plain. From an ecological perspective, the decrease in rainfall directly impacts all Cerrado communities by reducing water availability and indirectly by changing plant phenology/reproduction^42^ and alterations in interspecific interactions^43,44^. The environmental impacts of reduced rainfall in the Cerrado extend to aquatic ecosystems that declined 68,579 ha in their surface area between 1985 and 2021^45^, even with the creation of several large dam lakes for electricity production in this period (e.g., *Serra da Mesa* in 1996 with a surface area of 178,400 ha*, Manso* in 2000 with a surface area of 42,700 ha, *Serra do Falcão* in 2010 with a surface area of 21,884 ha, and *Estreito* in 2012 with a surface area of 40,000 ha). The expansion of the area occupied by dam lakes in Cerrado has not prevented recent episodes of a water crisis with water supply rationing to the population and temporary reduction/interruption of hydroelectric energy production. Furthermore, during severe drought events, the Brazilian population must also live with the sudden increase in food and energy prices which also affect other regions of the country^46^. Our results and projections of future scenarios generated by other studies also call into question the maintenance of the agricultural production model currently practiced in the Cerrado, particularly concerning the plantation system^38,47^. In recent years, studies have shown the relationship between agriculture expansion and streamflow reduction in Cerrado rivers, intensifying the debate between society and farmers in western Bahia^48,49^. As we demonstrate, this is one of the regions most affected by reduced rainfall in the Cerrado, and the water conflicts will probably become more frequent in the coming years. To conclude, we emphasize that the climate changes described here, especially the South Atlantic Subtropical Anticyclone strengthening, are causing the reduction of rainfall in Cerrado, which is more pronounced during the dry season and the beginning of the wet season.

## Methods

Our analyses were based on three independent datasets, which include direct observations (i.e., rainfall data) and the combination of model data with historical observations from satellites and conventional ground-based stations (i.e., ERA5 global reanalysis and TerraClimate). We have specifically chosen these datasets because they allowed us to compare the period before agricultural expansion in Cerrado and the first years of Hadley cell intensification with the present. In addition to precipitation data, we analyzed possible changes in atmospheric circulation, humidity, and evapotranspiration to elucidate the possible reasons for the reduction of total rainfall and frequency of rainy days on Cerrado. As mentioned earlier in the introduction section of this manuscript, we performed all analyses in four distinct periods according to the rainfall distribution patterns over Brazilian tropical areas throughout the year: the wet season (formed by December, January, February, and March), the beginning of the dry season (April and May), the dry season (June, July, August, and September), and the beginning of the wet season (October and November). We emphasize that data and analyses used in our study are open access and, therefore, could be applied in other Brazilian tropical ecoregions where climate changes are also taking place.

### Evaluation of trends in rainfall and frequency of rainy days at the local scale

To access historical precipitation records between 1960 and 2021, we used the Hidroweb portal (https://www.snirh.gov.br/hidroweb), which compiles the data collected by different Brazilian agencies. We selected 70 pluviometric stations with consistent data series (i.e., without lacking information for long periods) in localities spread across all Cerrado subregions. The list of 70 pluviometric stations and other information is summarized in Supplementary Table S3. We organized the daily rainfall (mm) records of each station according to the four periods considered, and the missing values of each locality were filled with the data observed at the nearest pluviometric station. We established two rules for filling in the missing values: (i) the number of missing records could not exceed 15% of the total data; (ii) the distance between stations should be less than 60 km (see details in Supplementary Table S4). Localities that did not meet these rules were excluded. In cases where the pluviometric station started recording data after 1960, we did not consider the previous years as missing values and analyzed only the period after the beginning of the data recording (see the period for each pluviometric station in Supplementary Table S3). After organizing the daily data for each location, we calculated both variables, the total rainfall (i.e., the sum of precipitation accumulated in each period) and frequency of rainy days (i.e., the sum of the number of days with precipitation events in each period). From data from 70 pluviometric stations, we calculated the monthly means of these two variables for the period 1960–1990. Then, we plotted these means values to demonstrate the annual precipitation cycle in the Brazilian Cerrado in the four periods considered in our analysis (Fig. 1b).

Then, we used Mann–Kendall test to analyze the local trends in both variables between 1960 and 2021 (Figs. 1, 2, 3, 4, 5a and b). This non-parametric method is useful for identifying temporal patterns that may not be immediately apparent, and for testing hypotheses about the presence or absence of trends^22^. Many researchers described the procedures of the Mann–Kendall test^23,24^. First, it is calculated the sign (i.e., direction of trends) for each pair of observations in the time series *x*_*i*_ ranked from *i* = *1* to *n–1* and *x*_*j*_ ranked from *j* = *i* + *1* to *n* data points, and each data observation x_i_ is used as a reference observation and is compared with all other data observations x_j_. Next, the Kendall’s S-statistics is computed from the sum of the signs and the variance of the S-statistics^24^. If the sum is positive, it suggests an upward trend in the data; if the sum is negative, it suggests a downward trend; and if the sum is close to zero, it suggests no trend. After that, a Z-test is used to determine the probability that the trend occurred by chance, given the sample size and the distribution of the data. If *|Zs|*> *(Zobs* = *Zα/2)* we can reject the null hypothesis, therefore indicating a significant trend at the *α* significance level, set to 5% (confidence level of 95%) in this stud. The Mann Kendall test is traditionally used in hydrological studies, especially for cases with high precipitation variance, such as the Cerrado^50^. The Mann–Kendall tests were performed using PAST.4.03. Lastly, we also quantified the changes in total rainfall and frequency of rainy days in each of the four periods by comparing the mean values of the last 31 years (1991–2021) to the 31 first years (1960–1990) for all 70 localities (Supplementary Figs. S1 and S2; Supplementary Tables S1 and S2). The same comparison for annual data is shown in Fig. 6d–e.

### Changes in rainfall, air humidity, and atmospheric circulation at the regional scale

We evaluated large-scale regional changes in total rainfall, air humidity, and atmospheric circulation based on the reanalysis ERA5-Land. This is a meteorological reanalysis project carried out by the European Centre for Medium -Range Weather Forecasts, and its data are provided directly on the Copernicus Climate Change Service website (https://cds.climate.copernicus.eu/cdsapp#!/dataset/reanalysis-era5-land-monthly-means?tab=overview). This product reports global data hourly with 9 km spatial resolution and covers the period from 1950 to the present^51^. At first, we analyzed the total precipitation data (mm/day), comparing the climate normals 1991–2021 and 1960–1990 for all four periods (Figs. 2, 3, 4, 5c) and annually (Fig. 6c). We performed a Student's t-test with 95% significance to compare the changes between the values of two climatic normals, and the results were shown using the GrADS application^52^. We also used the same approach to analyze the fields of relative humidity (%)(Figs. 2, 3, 4, 5e), specific humidity (g kg^-1^) (Figs. 2, 3, 4, 5f), U-component of wind (m s^-1^) (i.e., the zonal or eastward component of wind; Figs. 2, 3, 4, 5 g), V-component of wind (m s^-1^) (i.e., the meridional or northward component of wind; Figs. 2, 3, 4, 5 h), and vertical velocity of wind (Pa s^-1^) (Figs. 2, 3, 4, 5i). For the analysis of the fields of humidity (relative and specific) and horizontal winds (U and V-components), we have chosen to use the 850-hPa geopotential height due to its recognized importance for zonal and meridional moisture transport in the Cerrado^24,53^. For vertical velocity of wind (omega) analyses, we evaluated the changes between 1000 and 100 hPa in four meridians (i.e., −45°, −47.5°, −50°, and −52,5°) that cover almost the entire longitudinal extension of the Cerrado (Supplementary Fig. S3–S6). However, in the main figures of the manuscript, we have chosen to show the changes in omega only at 500-hPa geopotential height for the entire region to maintain the same pattern adopted for the other variables (Figs. 2, 3, 4, 5j). Finally, in the reanalysis of Sea Level Pressure (Supplementary Fig. S7), Vertically Integrated Moisture Flux, and 10 m Wind, we expanded the spatial scale of the study to include the entire South America/Atlantic Ocean domain in order to demonstrate the changes in SASA position and intensity. In both analyses, we applied the criterion that Fahad et al.^36^ used to represent the SASA position/extension through the 1020 hPa isobar. In Fig. 7, we overlapped the Vertically Integrated Moisture Flux and the intensity and direction of 10 m Wind in the same figure to demonstrate an integrated analysis and dynamic interaction of these two variables and their consequent impacts on seasonal rainfall regimes over Cerrado.

### Regional trends for evapotranspiration

Unfortunately, Brazilian meteorological agencies do not measure evapotranspiration in their weather stations. For this reason, we were forced to use data to access information about this variable in our study. The concept of the reference evapotranspiration assumes a reference grass surface across space, not short of water. Therefore, ET_o_ is a climatic parameter that evaluates the evaporative demand of the atmosphere independently of vegetation or crop type, crop development, soil, and management practices^33^. To explore trends in ET_o_, we used information from the TerraClimate dataset, which combines observations and models to provide global climate and climatic water balance climate variables for terrestrial surfaces in the period 1950–2021 at 1/24° (~ 4 km) spatial resolution^54,55^. TerraClimate evaluates monthly ET_o_ by applying the well-known Penman-Montieth approach. Comparisons via Pearson’s correlations between ET_o_ calculated in TerraClimate and data of 50 stations of the global network of FLUXNET were high, suggesting a good performance of the method, especially for non-forest ecosystems such as Cerrado^54^. We used the Google Earth Engine^56^, a cloud-based geospatial data analysis and visualization platform, to calculate the evapotranspiration trends between 1960 and 2021. We applied the Mann–Kendall test for all pixels included in the Cerrado area for all four periods evaluated (Figs. 2, 3, 4, 5d). To maintain consistency with the local analyzes of total rainfall and frequency of rainy days, we have chosen to display on the maps only the statistically significant trends with blue and red colors (for increasing and decreasing trends, respectively), while the gray color represented pixels with not-significant trends.

## Supplementary Information

Supplementary Information.

## Data Availability

The datasets used and/or analyzed during the current study available from the corresponding author on reasonable request.
