# Supplementary material for: Changes in atmospheric circulation and evapotranspiration are reducing rainfall in the Brazilian Cerrado
Source: Sci Rep. 2023 Jul 11;13:11236. doi: 10.1038/s41598-023-38174-x (PMC10336145; doi:10.1038/s41598-023-38174-x)
Supplement: Supplementary file 1 — Supplementary Information. [file 41598_2023_38174_MOESM1_ESM.pdf]

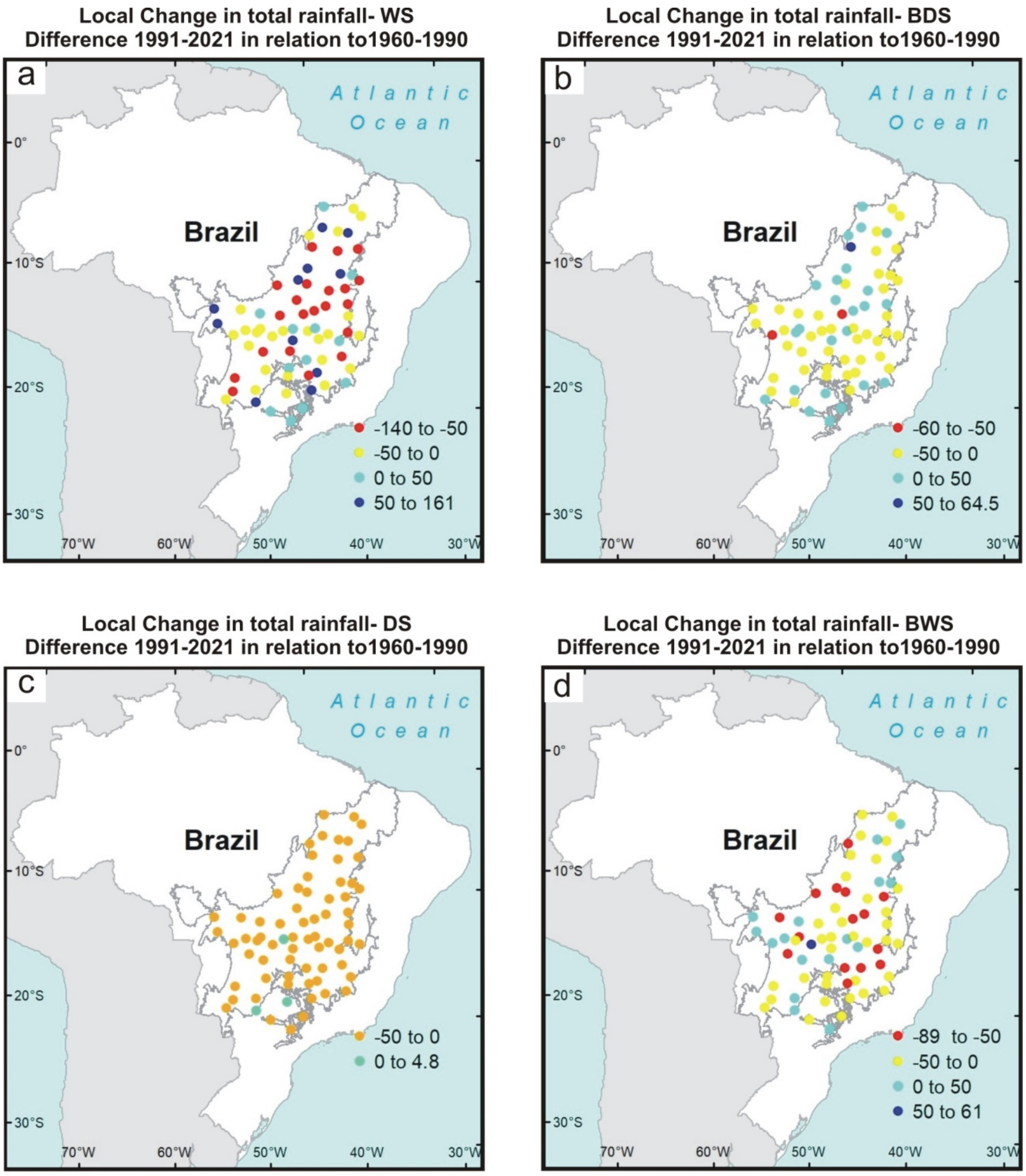

Supplementary Fig. S1. Local changes over the last six decades in total rainfall for all year periods. a─d, Local changes in total rainfall by comparing the climate normal 1991─2021 to the climate normal 1960─1990 in the wet season, the beginning of the dry season, the dry season, and the beginning of the wet season, respectively. The gray polygon show the official limits of the Brazilian Cerrado. Maps in the upper level were produced using ArcGIS (https://www.arcgis.com).

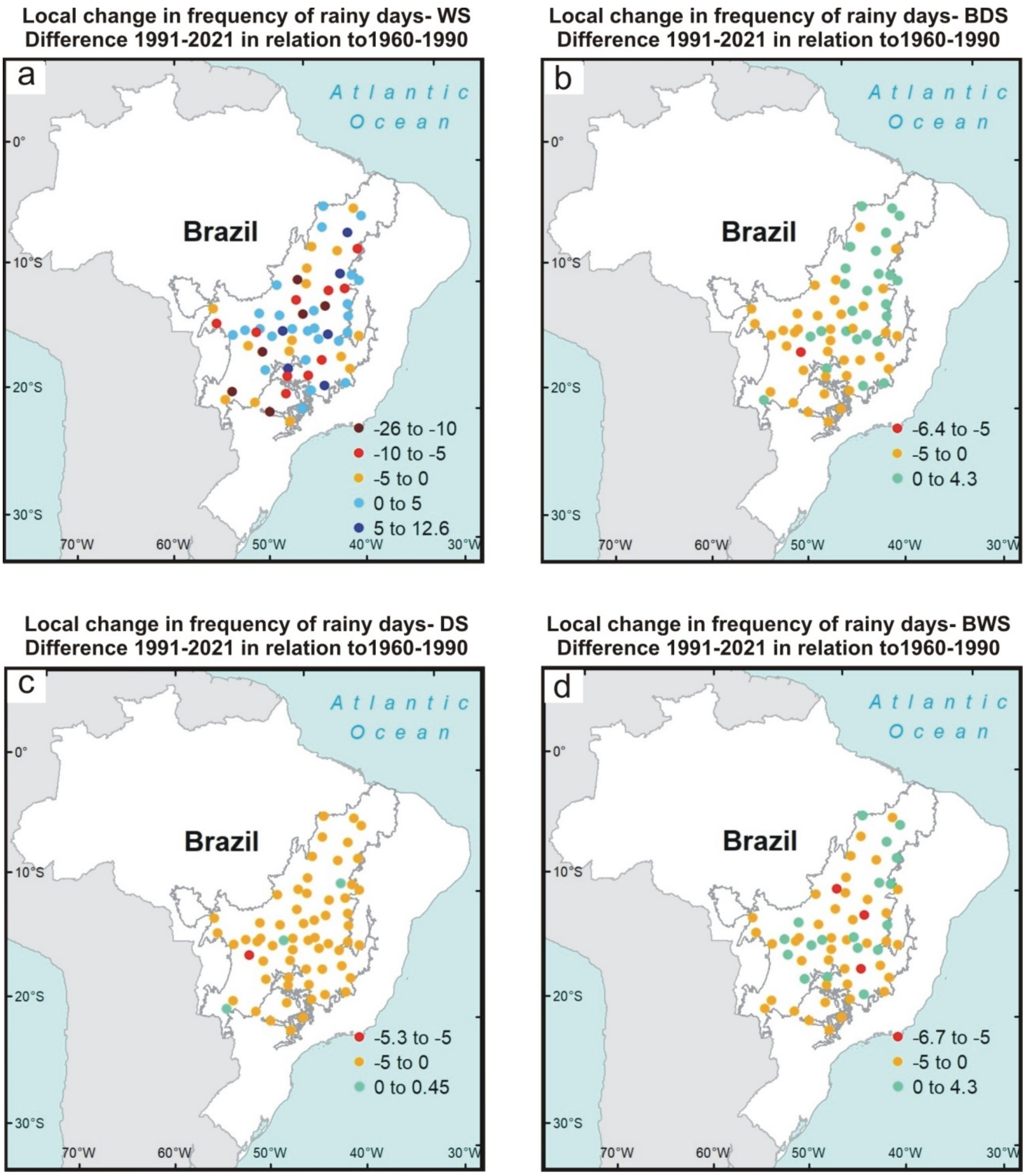

Supplementary Fig. S2. Local changes over the last six decades in frequency of rainy days for all year periods. a─d, Local changes in frequency of rainy days by comparing the climate normal 1991─2021 to the climate normal 1960─1990 in the wet season, the beginning of the dry season, the dry season, and the beginning of the wet season, respectively. The gray polygon show the official limits of the Brazilian Cerrado. Maps in the upper level were produced using ArcGIS (https://www.arcgis.com).

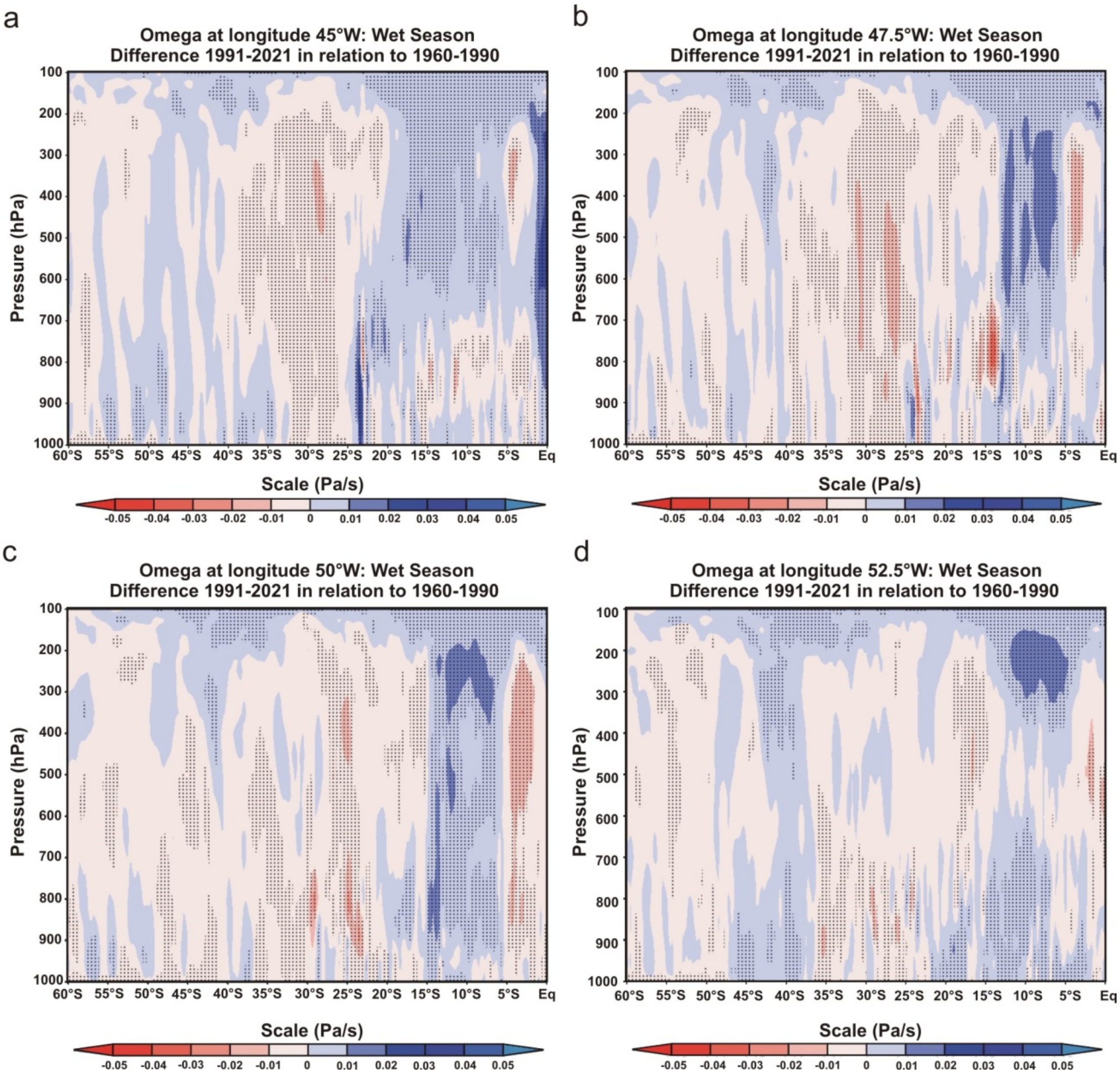

Supplementary Fig. S3. Changes in omega intensity between 1960-2021 during the wet season in the Brazilian Cerrado. a─d, Latitude-height cross sections of the vertical velocity of wind showing the changes in omega intensity (Pa/s) during the wet season in the meridians -45°, -47.5°, -50°, and -52,5°, respectively. The dotted areas represent locals with a significant difference ($p < 0.05$) between the climate normals 1991─2021 and 1960─1990.

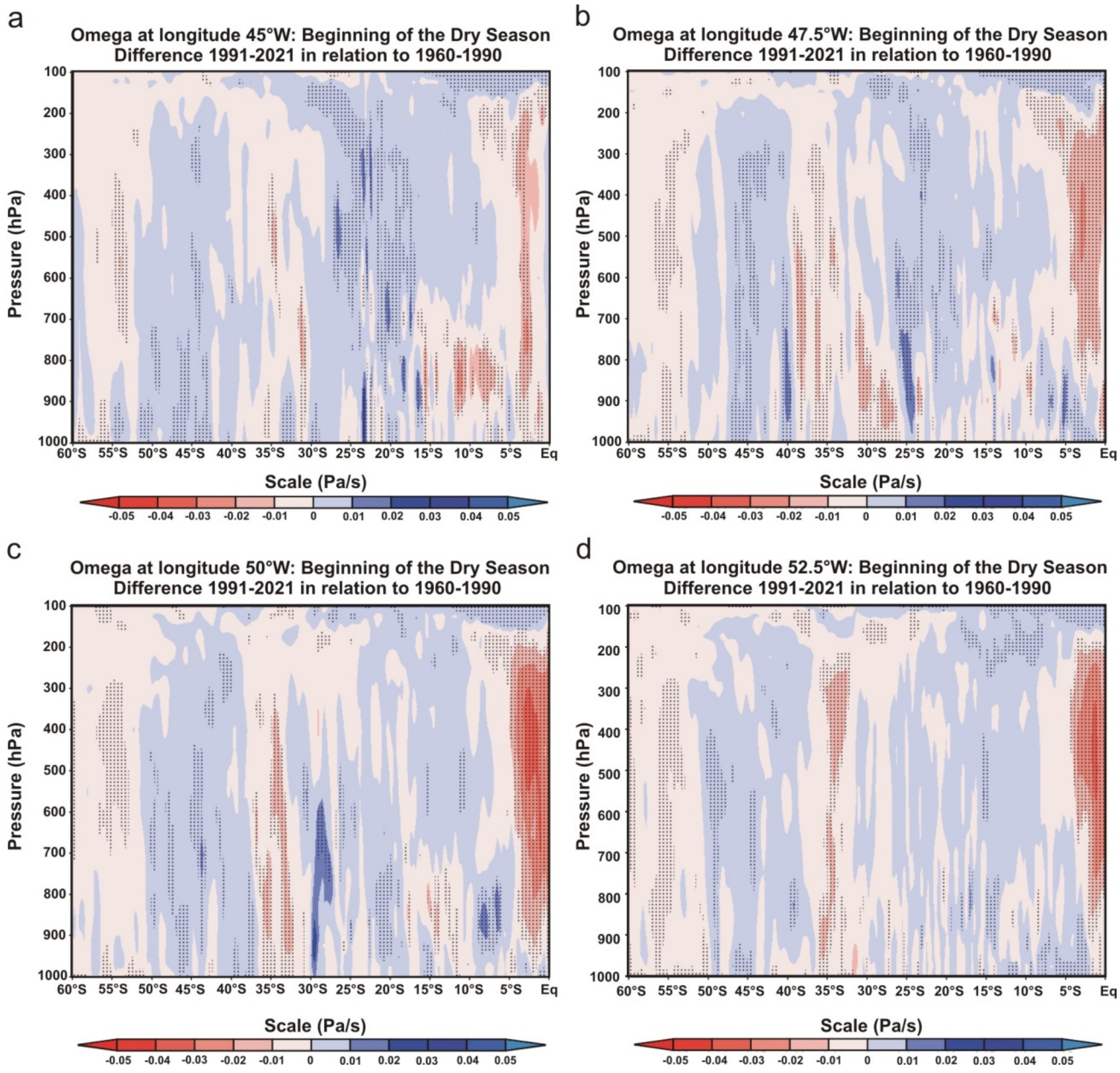

Supplementary Fig. S4. Changes in omega intensity between 1960-2021 during the beginning of dry season in the Brazilian Cerrado. a─d, Latitude-height cross sections of the vertical velocity of wind showing the changes in omega intensity (Pa/s) during the beginning of dry season in the meridians -45°, -47.5°, -50°, and -52,5°, respectively. The dotted areas represent locals with a significant difference ($p< 0.05$) between the climate normals 1991─2021 and 1960─1990.

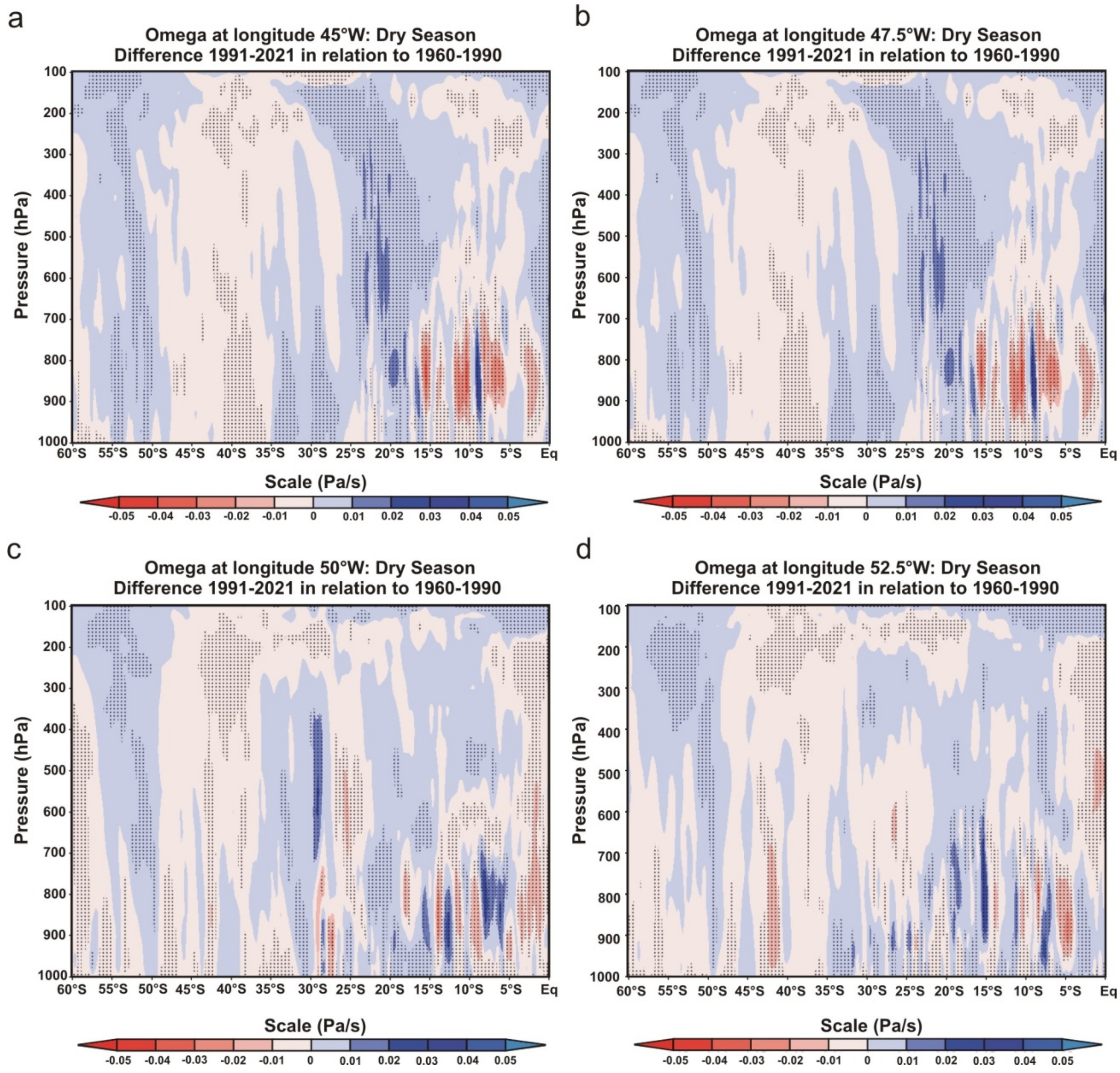

Supplementary Fig. S5. Changes in omega intensity between 1960-2021 during the dry season in the Brazilian Cerrado. a─d, Latitude-height cross sections of the vertical velocity of wind showing the changes in omega intensity (Pa/s) during the dry season in the meridians -45°, -47.5°, -50°, and -52,5°, respectively. The dotted areas represent locals with a significant difference ($p < 0.05$) between the climate normals 1991─2021 and 1960─1990.

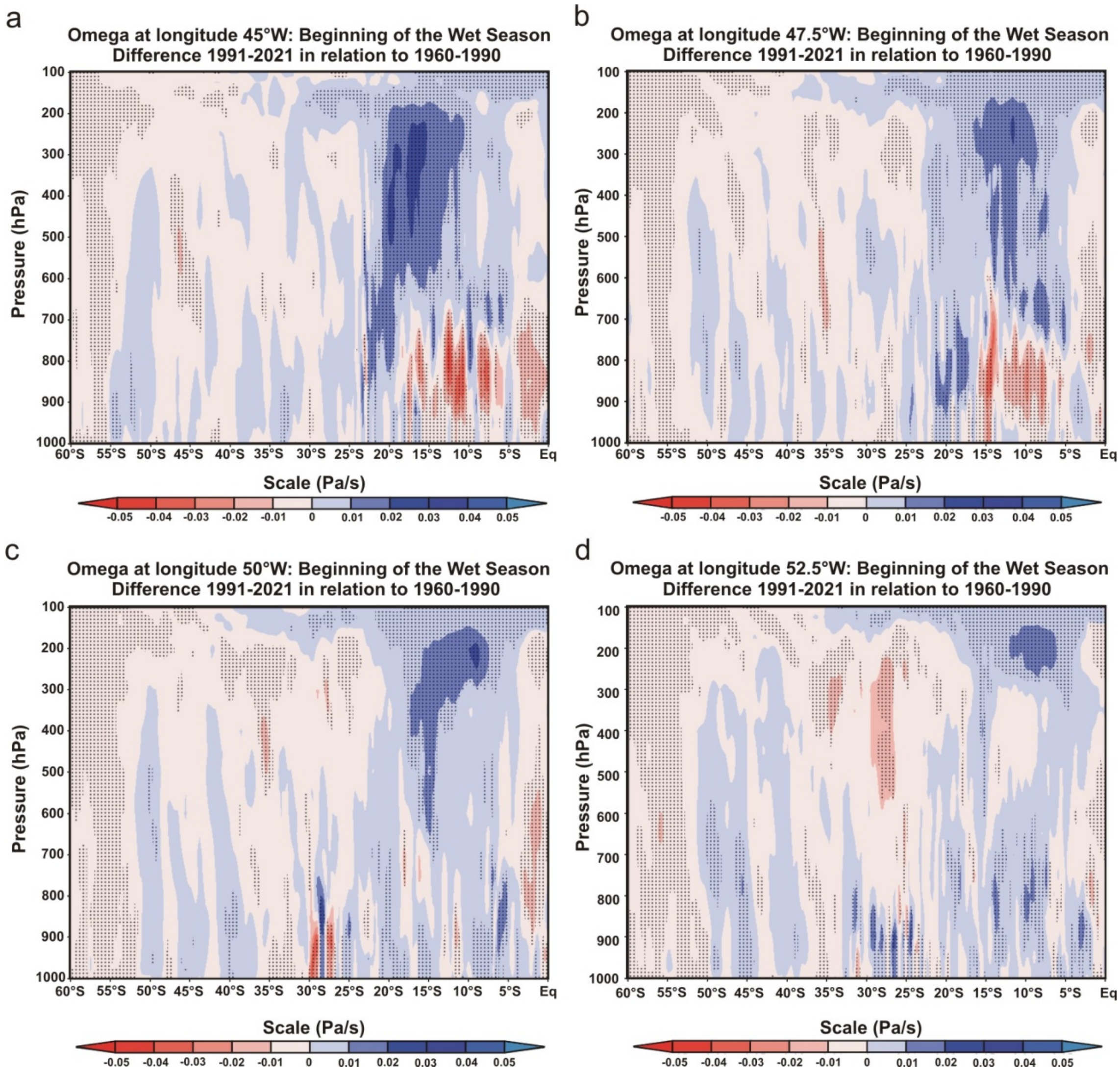

Supplementary Fig. S6. Changes in omega intensity between 1960-2021 during the beginning of wet season in the Brazilian Cerrado. a─d, Latitude-height cross sections of the vertical velocity of wind showing the changes in omega intensity (Pa/s) during the beginning of wet season in the meridians -45°, -47.5°, -50°, and -52,5°, respectively. The dotted areas represent locals with a significant difference ($p < 0.05$) between the climate normals v and 1960─1990.

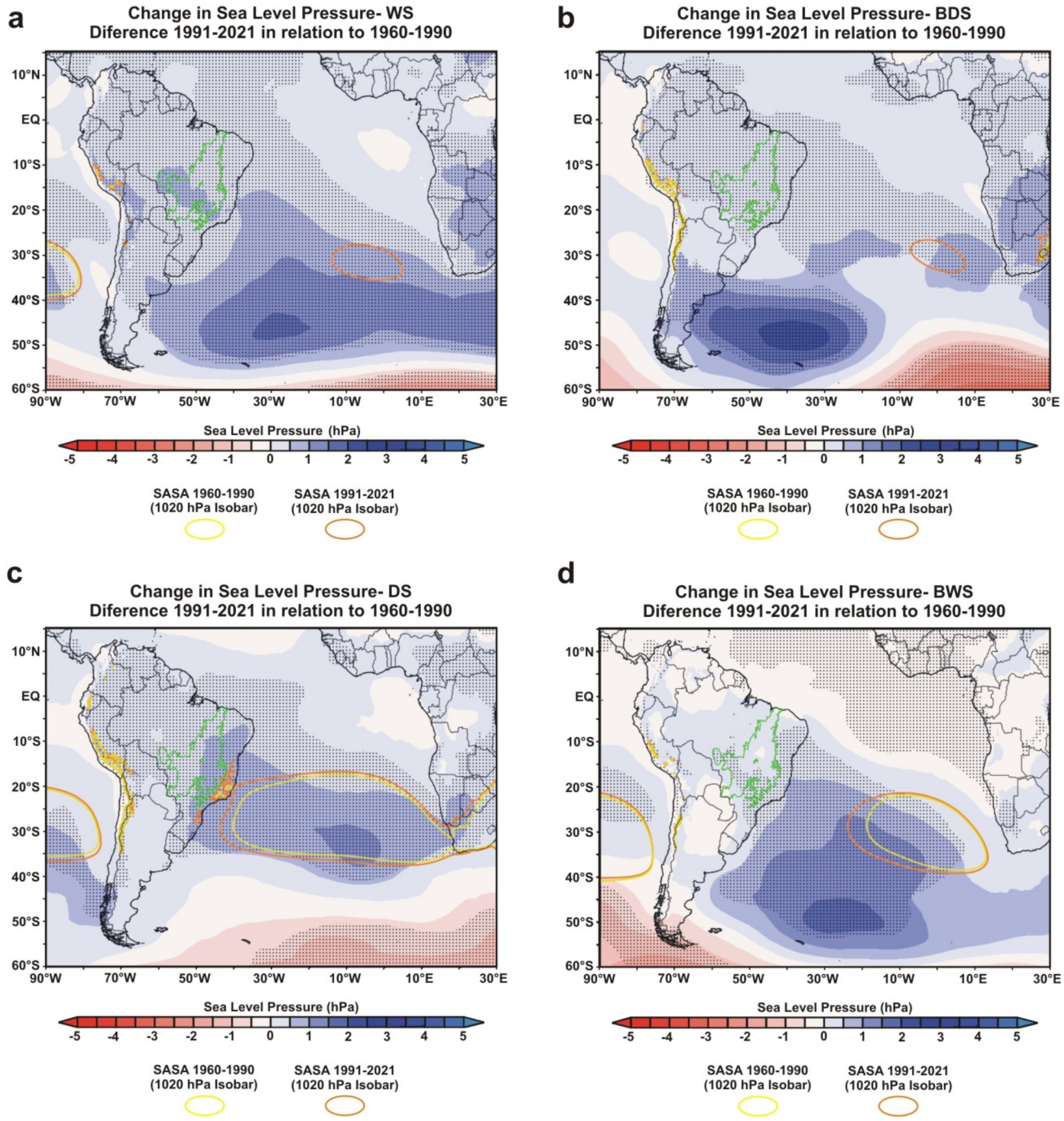

Supplementary Figure S7- Changes in Sea Level Pressure between 1960─2021 in the South America/South Atlantic domain. a-d, Sea Level Pressure changes recorded by ERA5 reanalysis in the Wet Season (WS), Beginning of Dry Season (BDS), Dry Season (DS), and Beginning of Wet Season (BWS), respectively. The dotted areas demonstrated a significant difference ($p < 0.05$) between 1991─2021 and 1960─1990 climate normals. The green polygon shows the official limits of the Brazilian Cerrado. The yellow and orange ellipses represent the 1020 hPa isobar and show the South Atlantic Subtropical Anticyclone (SASA) position/extension in 1960─1990 and 1991─2021, respectively. Maps in the upper level were produced using GrADS-Grid Analysis and Display System (http://opengrads.org).

Supplementary Table S1. Local changes in total rainfall over the last six decades in the Brazilian Cerrado. The absolute and proportional difference in total rainfall (mm) for each pluviometric station between 1991-2021 and 1960-1990. Where: WS- wet season; BDS- beginning of the dry season; DS- dry season; BWS- beginning of the wet season; Annual- total annual amount.

Pluviometric station	Absolute and proportional difference in Total rainfall (mm) between 1991-2021 and 1960-1990				
	WS	BDS	DS	BWS	Annual
Alto Araguaia	-38,9 (-3,9%)	-21,8 (-12,7%)	-25,8 (19,5	-54,3 (-13,8%)	-141 (-8,3%)
Alto Paraíso de Goiás	-140,6 (-14,7%)	6,3 (+4,6%)	-23,3 (-31,5%)	-89,3 (-21,1%)	-245 (-15,4%)
Alto Parnaíba	-113,4 (-11,9%)	-25,9 (-13,4%)	-25,8 (-58,1%)	-0,1 (0%)	-163,7 (-11,4%)
Aragarças	-36,5 (-3,6%)	6,8 (6,7%)	-43,3 (-44,5%)	-78,4 (-20,4%)	-150,8 (9,4%)
Arinos	-23,2 (-3,1%)	-19,2 (-19,7%)	-29,3 (61,9%)	-41,6 (-13,1%)	-111 (-9,1%)
Avaré	6,5 (+0,8%)	12,3 (+7,7%)	-6,3 (-2,5%)	4,7 (+2,1%)	22,7 (+1,6%)
Bady Bassit	-6,2 (-0,8%)	0,2 (+0,2%)	4,8 (+3,8%)	-24,4 (-9,3%)	-27,1 (-2%)
Balsas	-30,1 (-3,8%)	-19 (-10,5%)	-33,9 (-57,4%)	13,5 (6%)	-67,3 (-5,3%)
Bambuí	-4,2 (-0,5%)	10,5 (+8,7%)	-14,3 (-13,4%)	-35,3 (-10%)	-45,4 (-3%)
Bandeirantes	-101,1 (-10,6%)	-42,7 (-19,6%)	-46,3 (-19,9%)	-14,2 (-4,2%)	-210,7 (-12,1%)
Barra do Corda	-43,7 (-5,8%)	-28,5 (-11%)	-19,8 (-30,6%)	-7,2 (-5,6%)	-97,1 (-8,1%)
Barreiras	-68,2 (-9,4%)	-9,4 (-9,5%)	-27,3 (-67%)	-73,1 (-24,9%)	-175,9 (-15,2%)
Benjamin Barros	-61,2 (-6,2%)	-47,9 (-24,5%)	-41,5 (-30,9%)	1,3 (+0,5%)	-151,4 (-9,6%)
Boqueirão	-61 (-9,9%)	-12,1 (-11,9%)	-6,9 (-39,7%)	-21,9 (-10,5%0	-100 (-10,6%)
Brasília	-15,8 (-1,8%)	17,5 (+11,4%)	-23,9 (-29,1%)	0 (0%)	-22,7 (-1,5%)
Campina Verde	-44,7 (-5%)	-20,6 (-13,9%)	-15 (-13,8%)	-26,7 (-8,4%)	-107,5 (-7,3%)
Carolina	56,5 (+5,4)	13 (+3,5)	-27 (-28,9%)	-32,4 (-9,9%)	15,6 (+0,9%)
Catalão	19,4 (+2,0%)	-3,3 (-3%)	-13,7 (-18,9%)	-60,8 (-17,7%)	-60,8 (-4,1%)
Colinas do Maranhão	-39,8 (-5,1%)	-48,5 (-10,7%)	-24,2 (-33,5%)	15,3 (+10,4)	-98,1 (-7,6%)
Colinas do Tocantins	-0,9 (-0,1%)	38,5 (+14,2%)	-46,6 (-38,2%)	-59 (-14,2%)	-64,1 (-3,5%)
Correntina	-99,5 (-15,2%)	1,2 (+1,7%)	-11,3 (-58,3%)	-9,3 (-3,5%)	-117 (-11,7%)
Cristiano de Castro	-67,4 (-10,7%)	-29,5 (-20,2%)	-13,1 (-63,4%)	9,1 (+5,6%)	-100 (-10,4%)
Cuiabá	111,3 (+13,9%)	-21,6 (-11,8%)	-9,4 (-9,5%)	33,2 (+12,1%)	110,8 (+8,2%)
Diamantino	161,6 (+16,3%)	-9,9 (-5%)	-22,8 (-18,3%)	48,6 (+13,4%)	178,1 (+10,6%)
Fazenda Bom Jardim	104,6 (+16,9%)	-7,2 (-7,4%)	-7,1 (-39,5%)	29,5 (+15,25)	121,8 (+13,1%)
Fazenda Ingazeiro	-130,5 (-16,1%)	25,7 (26%)	-6,2 (-18,4%)	-58,6 (-17,8%)	-171,2 (-13,5%)
Formosa	8,3 (+0,9%)	-17,7 (-13,7%)	-20,9 (-34,9%)	-11,9 (-3,7%)	-41,6 (-3%)
Franca	54,7 (+5,5%)	-13,6 (-9,4%)	-16,1 (-11,4%)	-28,6 (-0,2%)	-3,2 (-0,2%)
Goiânia	62,2 (+6,5%)	12,4 (+8,0%)	-12,2 (-16,1%)	-22,1 (-5,7%)	40,7 (+2,6%)
Goiás Velho	-4,4 (-0,4%)	-19,1 (-13,1%0	3,5 (+6,2%)	-26,6 (-6,5%)	-51,9 (-2,9%)
Gouveia	-40,2 (-5,2%)	-10,1 (-10,5%)	-23,9 (-32,5%)	-31,6 (-9,3%)	-103,6 (-8,1%)
Gurupi	90,2 (+10,1%)	29,6 (+22,2%)	-28,1 (-45,8%)	-61,8 (22,7%)	30,7 (+2,3%)
Imperatriz do Maranhão	12,9 (+1,4%)	36,5 (+15,2%)	-27,9 (-32,9%)	-6,4 (-3,2%)	22,7 (+1,5%)
Iporá	-8,2 (-0,8%)	-2,1 (-1,7%)	-21,1 (-27,6%)	61 (+20,8)	27,6 (+1,8%)
Itajá	-49,2 (-5,1%)	-27,7 (-16,8%)	-18,9 (-15,3%)	-14,9 (-4,8%)	-111,6 (-7,2%)
Ituiutaba	32,1 (+3,8%)	-0,7 (-0,6%)	-19,8 (-21,1%)	-11,9 (-4%)	1,8 (+0,1%)
Janaúba	-29,3 (-5,8%)	-14,5 (-28,2%)	-15 (-64,1%)	-8,4 (-3,9%)	-66,2 (-8,3%)
Jaraguá de Goiás	12,2 (+1,1%)	-19,2 (-12%)	-24,3 (-31,2%)	-45,4 (-11,2%)	-77,9 (-4,6%)
Maracaju	-29,8 (-4,4%)	25,7 (+14,8%)	-7,9 (-3,2%)	-1,8 (-0,6%)	-7,9 (-0,6%)
Mozarlândia	-64,1 (-5,8%)	-12,2 (-9%)	-4,4 (-7,5%)	-14,8 (-4,3%)	-92 (-5,6%)
Niquelândia	-106,5 (9,6%)	-50,3 (-33,3%)	-22,2 (-33,6%)	-48,2 (-11,5%)	-234,7 (-13,4%)
Nova Xavantina	38,7 (+4%)	-28,4 (-22,6%)	-6 (-10%)	7,8 (+2,3%)	13,5 (+0,9%)
Novo Santo Antônio	-140,8 (-13,2%)	3,7 (+3%)	-25,1 (-50%)	-63,5 (-15,8%)	-225,5 (-13,7%)
Paraguaçu Paulista	6,2 (+0,9%)	31,6 (+22,2%)	-0,7 (-0,3%)	-28,7 (-9,9%)	15,8 (+1,1%)
Paranatinga	-36,7 (-3,2%)	-17,1 (-10,1%)	-44,6 (-37,7%)	-54,1 (-12,5%)	-154,3 (-8,2%)
Pedras de Maria Cruz	-65,3 (-10,6%)	-15,1 (-28,1%)	-17,8 (-64,4%)	-14,3 (-5,6%)	-111,9 (-11,8%)
Pedro Afonso	-54 (-5%)	64,5 (+34,5%)	-28,9 (-35,5%)	-42 (-11,1%)	-56,7 (-3,3%)
Pedro Leopoldo	43,7 (+5,4%)	4,4 (+7%)	-7 (-10,2%)	-44,2 (-13,2%)	-2,4 (-0,2%)
Peixe	-90,1 (-8,8%)	-5,2 (-3,4%)	-17,7 (-32,8%)	-61 (-17,8%)	-172 (-10,9%)
Pontalina	-53,6 (-5,8%)	-19,8 (-28,4%)	-14,6 (19,7%)	21,5 (+7,3%)	-74,4 (-5,2%)
Ponte Firme	-27,8 (-2,8%)	-2,1 (-2,3%)	-11,9 (-22,2%)	-63,9 (-17,5%)	-105,6 (-7%)
Porangatu	-62,9 (-5,8%)	0,9 (+0,8%)	-30 (-45,8%)	-12,1 (-3,5%)	-103,2 (-6,4%)
Porto Nacional	91,1 (+9%)	49,9 (27%)	-10,9 (-18%)	-11,8 (-3,3%)	121,4 (+7,6%)
Porto Uerê	84,8 (+12.8%)	-20,4 (-11,2%)	2,2 (+1,15)	33,5(+14,6%)	97,1 (+7,6%)
Porto Velho	-11,9 (-1,6%)	6,3 (+4,4%)	-18 (-10,5%)	2 (+0,8%)	-23,1 (-1,8%)
Ribas do Rio Pardo	-0,5 (-0,1%)	-16,9 (-9,2%)	-8,7 (-4,9%)	-4,9 (-1,6%)	-34,2 (-2,4%)
Ribeiro Gonçalves	68,1 (+10,5)	17,4 (+11,4)	-7,3 (-23%)	-16,7 (-8,6%)	65,5 (+6,4%)
Rondonópolis	-6,3 (-0,7%)	-60,4 (-35,1%)	-11,8 (-11,5%)	17,7 (+6,2%)	-68,7 (-4,8%)
Salitre	70,5 (+8,5%)	-4,5 (-4%)	-18,7 (-17,1%)	-48,4 (-13,3%)	-1,8 (-0,1%)
Santa Juliana	-120,1 (-11,6%)	-1,1 (-0,9%)	-11,3 (-11,5%)	-84,4 (-21,6%)	-215,5 (-13,1%)
Santa Rita de Cássia	0,3 (0,1%)	-12,2 (-11,6%)	-9,9 (-50,9%)	12,6 (+5,8%)	-8,5 (-0,9%)

São Carlos	35,3 (+4%)	17,2 (+13,6%)	-3,8 (-2,5%)	-1,4 (-0,5%)	48,2 (+3,3%)
São Gonçalo	-19,8 (-3,3%)	-11,8 (-17,3%)	-2,4 (-13%)	-9 (-3,6%)	-41,5 (-4,4%)
São Romão	37 (+5,8%)	-8,4 (12,8%)	-6,8 (25,8%)	-53,3 (-18,9%)	-30,4 (-3%)
Taguatinga	-98,2 (-8,6%)	24 (+14,6%)	-25,4 (-52,7%)	-38,3 (-9,7%)	-137,1 (-7,8%)
Tesouro	-25,7 (-2,2%)	-25 (-14,1%)	-49,6 (-38,4%)	0,7 (+0,2%)	-98,3 (-5,3%)
Torixoreu	-49,1 (-5,3%)	23,8 (27,9%)	-19,8 (-24,6%)	-37,1 (-12,5%)	-82,1 (-5,9%)
Unaí	-18,2 (-2,2%)	-14,3 (-12,7%)	-15,8 (-30,5%)	1,3 (+0,4%)	-44,4 (-3,4%)
Varzea da Palma	-140,1 (-17,7%)	-36,8 (-43,9%)	-21,5 (-45%)	-63,8 (-19,4%)	-261,2 (-20,8%)
Vau do Balsamo	-111,1 (-14,7%)	-21,6 (-10,4%)	-11 (-4,9%)	-47,6 (-15,4%)	-197,2 (-13,2%)

Supplementary Table S2. Local changes in frequency of rainy days over the last six decades in the Brazilian Cerrado. The absolute and proportional difference in total rainfall (mm) for each pluviometric station between 1991-2021 and 1960-1990. Where: WS- wet season; BDS- beginning of the dry season; DS- dry season; BWS- beginning of the wet season; Annual- total annual amount.

Pluviometric station	Absolute and relative difference in number of rainy days between 1991-2021 and 1960-1990				
	WS	BDS	DS	BWS	Annual
Alto Araguaia	-0.7 (-0.9%)	-2.6 (-16.7%)	-5.3 (37.7%)	0.3 (1%)	-8.4 (-6.4%)
Alto Paraíso de Goiás	2.3 (+3.7%)	2.7 (+22.7%0	-2.4 (-32.5%)	-3.1 (-10.3%)	-0.5 (-0.4%)
Alto Parnaíba	-1.6 (-2.2%)	1.6 (+9.7%)	-2.1 (-37.5%)	-13.2 (-3.4%)	-5.5 (-4.6%)
Aragarças	1.1 (+1.6%)	-1.5 (-13.4%)	-3.4 (-34.6%)	-1.2 (-4.7%)	-5 (-4.3%)
Arinos	6.7 (+13.9%)	1 (+14.4%)	-1.2 (-26.2%)	-0.7 (-3.1%)	5.8 (+6.9%)
Avaré	-2.9 (-5.3%)	-1.6 (-11.6%)	-4.4 (-18.8%)	-0.7 (-4.1%)	-9.8 (-8.8%)
Bady Bassit	-7.4 (-14.9%)	-1.7 (-18.4%)	-2.4 (-20.3%)	-4 (-22.1%)	-15.5 (-17.4%)
Balsas	NA	NA	NA	NA	NA
Bambuí	6.4 (+11%)	1.7 (+15.4%)	-0.8 (-6%)	1.1 (+4.4%)	8.4 (+7.9%)
Bandeirantes	NA	NA	NA	NA	NA
Barra do Corda	-1.5 (-2.3%)	0.2 (+0.7%)	-2.1 (-18.3%)	-2.8 (-18.8%0	-6.3 (-5.3%)
Barreiras	-7.7 (-12.9%)	-2.5 (-18%)	-2.8 (-47.9%)	-6.7 (-25.7%)	-19.7 (-18.7%)
Benjamin Barros	-11.1 (-14%)	-6.4 (-33.7%)	-4.6 (-34.4%)	-4 (-16.3%)	-26 (-19.1%)
Boqueirão	2.3 (+5.5%)	0.5 (5.5%)	-0.8 (-31.3%)	0 (0)	1.9 (+1.9%)
Brasília	4.6 (+6.5)	0.6 (+4.4%)	-1.3 (-13.4%)	-0.2 (-2.9%)	3.8 (+2.9%)
Campina Verde	-6.5 (-9.9%)	-3.3 (-24.7%)	-4.5 (-32.8%)	-1.5 (-6.8%)	-15.9 (-13.8%)
Carolina	1.2 (1.6%)	-2.1 (-6.8%)	-3 (-26.8%)	-2.9 (11.5%)	1 (+0.7%)
Catalão	0.2 (+0.2%)	-0.4 (-3.2%)	-1.9 (-19.1%)	-0.9 (-3.3%)	-3.1 (2.6%)
Colinas do Maranhão	0.6 (+1.1%)	1.2 (+3.6%)	-2 (-24.3%)	0.7 (+5.4%)	-0.2 (-0.2%)
Colinas do Tocantins	NA	NA	NA	NA	NA
Correntina	0.4 (+1%)	+0.6 (+9.5%)	-1 (30%)	-0.8 (-4.1%)	-0.8 (-1.1%)
Cristiano de Castro	-6.5 (-14.7%)	-2.8 (-22.6%)	-0.4 (-17.9%)	0.5 (+4.4%)	-9.2 (-13.1%)
Cuiabá	-7 (-9.6%)	-3.1 (-18.2%)	-3.3 (-27.4%)	-4.1 (-16.9%)	-17.5 (-13.5%)
Diamantino	-0.9 (-1.3%)	-0.8 (-5%)	-0.3 (-3.8%)	-1 (-3.7%)	-3 (-2.5%)
Fazenda Bom Jardim	5.7 (+16.4%)	2.5 (+43.1%)	0 (0)	4.3 (+12.6%)	12.6 (+23.3%)
Fazenda Ingazeiro	-11.9 (-20.9%)	-1.1 (-12.6%)	-1.2 (-31.6%)	-6.2 (-26.1%)	-20.4 (-21.8)
Formosa	0.2 (+0.2%)	-0.1 (-0.8%)	-1.8 (-25.3%)	0.8 (+2.8%)	-1 (-0.9%)
Franca	0.1 (+0.1%)	-2 (-13.8%)	-3.4 (-20.6%)	-1.6 (-5.8%)	-6.9 (-5.3%)
Goiânia	-1 (-1.3%)	-0.7 (-4.7%)	-2.4 (-21.8%)	-1.1 (-3.3%)	-5.3 (-3.8%)
Goiás Velho	6.7 (+9.1%)	0.4 (+3.6%)	0.5 (+6.4%)	2.4 (+8.6%)	10 (+8.3%)
Gouveia	-3.6 (-6.4%)	-0.2 (-2.1%)	-1.4 (-13.3%)	-4.3 (-15.8%)	-9.4 (-8.9%)
Gurupi	-11.3 (-17.8%)	-2.7 (-21.6%)	-3 (-52.8%)	-5.4 (-25.7%)	-22.4 (-21.8%)
Imperatriz do Maranhão	0 (0)	2.6 (+11.6%)	-2.4 (-22%)	0.1 (+0.5%)	0.3 (0.3%)
Iporá	3.3 (+5%)	1.2 (+12.7%)	-0.8 (-11%)	2.2 (+9.1%)	5.9 (+5.5%)
Itajá	1.6 (+2.9%)	-2.3 (-20.2%)	-1.6 (-14.2%)	0.6 (+2.9%)	-1.7 (-1.8%)
Ituiutaba	9.1 (+17%)	1.3 (+15%)	-0.7 (-8%)	2 (+9.7%)	11.7 (+12.7%)
Janaúba	-0.8 (-2%)	-0.2 (-5%)	-2.3 (-64.3%)	-2.7 (-14.7%)	-6 (-9.4%)
Jaraguá de Goiás	1.7 (+2.7%)	-0.9 (-8.4%)	-2.9 (-37.9%)	-2.2 (-9.3%)	-4.4 (-4.4%)
Maracaju	-1.2 (-2.8%)	1 (+9%)	0.2 (+1.2%)	-0.1 (-0.3%)	-0.1 (-0.1%)
Mozarlândia	3.6 (+5.2%)	-0.2 (-1.4%)	-2.2 (-28.6%)	0.3 (+1%)	1.4 (+1.3%)
Niquelândia	-10 (-13.5%)	-4.6 (-31.8%)	-4.1 (-50.7%)	-6.7 (-21.3%)	-25.4 (-19.8%)
Nova Xavantina	3.6 (+5.2%)	-0.2 (-1.4%)	-2.2 (-28.6%)	0.3 (+1%)	1.4 (+1.3%)
Novo Santo Antônio	2 (+4%)	-0.4 (-4.3%)	-1.4 (-33%)	-0.5 (-2.8%)	-0.4 (-0.4%)
Paraguaçu Paulista	-10.4 (-19.7%)	-1.6 (-14.2%)	-4.8 (-23.1%)	-3.5 (-18.7%)	-20.1 (-19.4%)
Paranatinga	NA	NA	NA	NA	NA
Pedras de Maria Cruz	1.5 (+3.8%)	-0.4 (-7.2%)	-1.4 (-34.5%)	-0.9 (-4.9%)	-1.2 (-1.8%)
Pedro Afonso	-1.9 (-2.3%)	4.3 (+23.9%)	-2.8 (-28.9%)	-1.6 (-5.4%)	-1.9 (-1.3%)
Pedro Leopoldo	3.1 (+6.3%)	1.9 (+28.5%)	-0.2 (-1.9%)	-1.5 (-6.5%)	3.4 (+3.8%)
Peixe	-3.2 (-4.2%)	1.4 (+10%)	-3.3 (-46.6%)	-1.5 (-5.8%)	-6.6 (-5.4%)
Pontalina	-2.1(11.2%)	-1.5 (-13.2%)	-3 (-31.4%)	-0.1 (-0.3%)	-6.8 (-6.3%)
Ponte Firme	-7 (-12.8%)	-0.7 (-0.7%)	-1 (-20%)	-5.8 (24.4%)	-14.5 (-15.8%)
Porangatu	-6.2 (-9.8%)	-1.6 (-15.4%)	-2.3 (-43.4%)	-1.9 (-8.5%)	-11.9 (-11%)
Porto Nacional	-2 (-2.5%)	1.5 (7.9%)	-1.5 (-22.3%)	-2.3 (-8%)	-4.2 (-3.2%)
Porto Uerê	-1.9 (-5.2%)	-1.7 (-17.1%)	-1.6 (-12%)	-1.1 (-8%)	-6.3 (-8.5%)
Porto Velho	NA	NA	NA	NA	NA
Ribas do Rio Pardo	-10.6 (-18.5%)	-4.4 (-32.7%)	-4 (-25.8%)	-1.5 (-7.7%)	-20.5 (-19.4%)
Ribeiro Gonçalves	5.3 (+13.7%)	2.6 (+26.1%)	-1.4 (-39.8)	2.1 (+23%)	8.7 (14.1%)
Rondonópolis	0.7 (+1.1%)	-2.7 (-21.4%)	-2.3 (-23.2%)	-0.1 (-0.6%)	-4.4 (-4.1%)
Salitre	NA	NA	NA	NA	NA
Santa Juliana	-9.5 (-13.6%)	-2.9 (-22.6%)	-2.6 (-22.8%)	-4.8 (-17.8%)	-19.9 (-16.4%)
Santa Rita de Cássia	3.9 (+8.7%)	1.1 (+12.1%)	-0.5 9-22.4%)	0.6 (+3.8%)	5.2 (+7%)

São Carlos	2.5 (+4%)	-0.9 (-6.3%)	-1 (5.7%)	-1 (-4%)	0 (0)
São Gonçalo	2.5 (+5.9%)	0.5 (+7.7%)	-0.2 (-9.2%)	1 (+5.1%)	3.7 (+5.3%)
São Romão	4.8 (+11%)	0.4 (+6.5%)	-0.1 (-3.3%)	1.1 (+5.6%)	6.2 (8.6%)
Taguatinga	-6.6 (-8.4%)	0.5 (+3.2%)	-2.5 (-43.5%)	-4.1 (-13.5%)	-12.7 (-9.8%)
Tesouro	2.4 (+3.7%)	-0.9 (7.7%)	-2.4 (25.6%)	3.1 (+14.7%)	2.5 (+2.3%)
Torixoreu	-5.7 (-8.9%)	-2.1 (-21.9%)	-2.5 (-33%)	-3.8 (-16.3%)	-14 (-13.5%)
Unaí	4.9 (+9.1%)	0.6 (+6.8%)	-0.8 (-14.4%)	0.2 (+0.9%)	4.9 (+5.3%)
Varzea da Palma	-4.5 (-9.2%)	-1.1 (-15.1%)	-1.3 (-24.8%)	-2.8 (-12.2%)	-9.8 (-11.5%)
Vau do Balsamo	-11.8 (-21%)	-3.9 (-26.6%)	-3.8 (-20.3%)	-3.3 (-16.1%)	-22.8 (-20.7%)

Supplementary Table S3. Identification and location of pluviometric stations used in this study. List of pluviometric stations and their respective latitude, longitude, state agency, and data period analyzed in the study. Where: INMET- Instituto Nacional de Meteorologia; CPRM- Companhia de Pesquisa de Recursos Minerais; DAEE-SP- Departamento de Águas e Energia Elétrica do Estado de São Paulo.

Pluviometric station	Latitude	Longitude	Agency	Period analyzed
Alto Araguaia	-17,3	-53,22	CPRM	1969-2021
Alto Paraíso de Goiás	-14,13	-47,51	CPRM	1969-2021
Alto Parnaíba	-9,1	-45,93	INMET	1977-2021
Aragarças	-15,90	-52,24	INMET	1971-2021
Arinos	-15,92	-46,12	CPRM	1963-2021
Avaré	-23,1	-48,92	DAEE-SP	1960-2021
Bady Bassit	-20,92	-49,45	DAEE-SP	1960-2021
Balsas	-7,53	-46,03	INMET	1977-2021
Bambuí	-20,02	-45,96	CPRM	1960-2021
Bandeirantes	-19,91	-54,35	CPRM	1976-2021
Barra do Corda	-5,5	-44,93	INMET	1970-2021
Barreiras	-12,15	-45,01	INMET	1973-2021
Benjamin Barros	-17,70	-51,89	CPRM	1974-2021
Boqueirão	-11,36	-43,85	CPRM	1960-2021
Brasília	-15,78	-47,92	INMET	1963-2021
Campina Verde	-19,54	-49,48	CPRM	1976-2021
Carolina	-7,33	-47,46	INMET	1962-2021
Catalão	-18,15	-47,96	INMET	1961-2021
Colinas do Maranhão	-6,03	-44,23	CPRM	1969-2021
Colinas do Tocantins	-8,05	-48,48	CPRM	1972-2021
Correntina	-13,34	-44,65	CPRM	1972-2021
Cristiano de Castro	-8,81	-44,21	CPRM	1963-2021
Cuiabá	-15,62	-56,11	INMET	1960-2021
Diamantino	-14,41	-56,45	INMET	1969-2021
Fazenda Bom Jardim	-10,99	-10,99	CPRM	1978-2021
Fazenda Ingazeiro	-13,69	-46,57	CPRM	1969-2021
Formosa	-15,54	-47,33	INMET	1974-2021
Franca	-20,52	-47,20	DAEE-SP	1960-2021
Goiânia	-16,67	-49,26	INMET	1961-2021
Goiás Velho	-15,94	-50,14	INMET	1961-2021
Gouveia	-18,46	-43,74	CPRM	1960-2021
Gurupi	-11,74	-49,14	CPRM	1972-2021
Imperatriz do Maranhão	-5,54	-47,48	INMET	1977-2021
Iporá	-16,42	-51,12	CPRM	1974-2021
Itajá	-19,14	-51,53	CPRM	1973-2021
Ituiutaba	-18,94	-49,46	CPRM	1968-2021
Janaúba	-15,77	-43,27	CPRM	1970-2021
Jaraguá de Goiás	-15,76	-49,34	CPRM	1965-2021
Maracaju	-21,61	-55,13	CPRM	1973-2021
Mozarlândia	-14,74	-50,58	CPRM	1974-2020
Niquelândia	-14,48	-48,46	CPRM	1970-2021
Nova Xavantina	-14,67	-52,36	CPRM	1969-2021
Novo Santo Antônio	-12,29	-50,96	CPRM	1970-2021
Paraguaçu Paulista	-22,42	-50,87	DAEE-SP	1960-2020
Paranatinga	-14,42	-54,05	CPRM	1974-2021
Pedras de Maria Cruz	-15,6	-44,39	CPRM	1973-2021
Pedro Afonso	-8,97	-48,18	INMET	1978-2021
Pedro Leopoldo	-19,63	-44,05	CPRM	1960-2021
Peixe	-12,02	-48,35	INMET	1976-2021
Pontalina	-17,51	-49,44	CPRM	1974-2021
Ponte Firme	-18,03	-46,41	CPRM	1960-2021
Porangatu	-13,41	-49,16	CPRM	1974-2021
Porto Nacional	-10,76	-48,42	INMET	1960-2021
Porto Uerê	-21,73	-52,33	CPRM	1973-2021
Porto Velho	-20,80	-52,38	CPRM	1973-2021
Ribas do Rio Pardo	-20,44	-53,75	CPRM	1973-2021
Ribeiro Gonçalves	-7,56	-45,24	CPRM	1962-2021
Rondonópolis	-16,47	-54,66	CPRM	1971-2021
Salitre	-19,07	-46,79	CPRM	1967-2021
Santa Juliana	-19,31	-47,52	CPRM	1960-2021
Santa Rita de Cássia	-11,00	-44,52	INMET	1960-2021
São Carlos	-21,98	-47,88	INMET	1961-2021

São Gonçalo	-14,31	-44,46	CPRM	1960-2021
São Romão	-16,37	-45,08	CPRM	1960-2021
Taguatinga	-12,40	-46,41	INMET	1974-2021
Tesouro	-16,08	-53,55	CPRM	1972-2021
Torixoreu	-16,2	-52,55	CPRM	1975-2021
Unaí	-16,35	-46,89	CPRM	1965-2021
Varzea da Palma	-17,59	-44,70	CPRM	1960-2021
Vau do Balsamo	-20,99	-54,50	CPRM	1973-2021

Supplementary Table S4. Identification of alternative pluviometric stations used to fill in missing values from the main pluviometric stations. List of pluviometric stations and their respective missing values amount (%), alternative pluviometric station, state agency, and distance between pluviometric stations (km). Where: INMET- Instituto Nacional de Meteorologia; CPRM- Companhia de Pesquisa de Recursos Minerais; DAEE-SP- Departamento de Águas e Energia Elétrica do Estado de São Paulo; CEMADEN- Centro Nacional de Monitoramento e Alertas de Desastres Naturais; CODEVASF- Companhia de Desenvolvimento dos Vales do São Francisco e do Parnaíba

Pluviometric station	Missing values	Alternative pluviometric station	Agency	Distance between pluviometric stations
Alto Araguaia	2%	Fazenda Babilônia	CPRM	13 km
Alto Paraíso de Goiás	4,5%	Alto Paraíso de Goiás	INMET	<5 km
Alto Parnaíba	8,5%	Alto Parnaíba	CPRM	<5 km
Aragarças	7,6%	Bom Jardim de Goiás	CPRM	35 km
Arinos	0,4%	Arinos	INMET	< 5km
Avaré	1,9%	Cerqueira Cesar	DAEE-SP	25 km
Bady Bassit	1,5%	São José do Rio Preto	DAEE-SP	12 km
Balsas	5%	Balsas	CEMADEN	< 5km
Bambuí	1,4%	Bambuí	INMET	< 5km
Bandeirantes	14,4%	Jaguarí	CPRM	21 km
Barra do Corda	0,5%	Flores	CPRM	34 km
Barreiras	4,7%	Barreiras	CODEVASF	<5 km
Benjamin Barros	3,1%	Jataí	INMET	25 km
Boqueirão	0,2%	Fazenda Macambira	CPRM	43 km
Brasília	0%	–	–	–
Campina Verde	0,5%	Campina Verde	INMET	< 5km
Carolina	0%	–	–	–
Catalão	3,7%	Três Ranchos	CPRM	26 km
Colinas do Maranhão	0,6%	Colinas	INMET	<5 km
Colinas do Tocantins	0,1%	Fazenda Primavera	CPRM	45 km
Correntina	8,6%	Correntina	INMET	<5 km
Cristiano de Castro	8,2%	Bom Jesus do Piauí	INMET	31 km
Cuiabá	4,1%	N.S^{a}. do Livramento	CPRM	26 km
Diamantino	2,5%	Nortelândia	CPRM	40 km
Fazenda Bom Jardim	2,1%	Formosa do Rio Preto	CPRM	37 km
Fazenda Ingazeiro	5,5%	Posse	INMET	47 km
Formosa	1,3%	Taquara	CAESB	20 km
Franca	6,9%	Franca	INMET	<5 km
Goiânia	0,8%	Trindade	CPRM	22 km
Goiás Velho	5,6%	Itaberaí	CPRM	35 km
Gouveia	3,3%	Usina Parauna	CPRM	31 km
Gurupi	1,8%	Gurupi	INMET	<5 km
Imperatriz do Maranhão	4,2%	Buritirama	CPRM	51 km
Iporá	3,8%	Israelândia	CPRM	25 km
Itajá	2%	Cassilândia	INMET	19 km
Ituiutaba	3,7%	Ituituva	INMET	< 5km
Janaúba	0,6%	Janaúba	INMET	< 5km
Jaraguá de Goiás	2,4%	Pirenópolis	INMET	40 km
Maracaju	2,7%	Maracaju	CEMADEN	< 5km
Mozarlândia	0,8%	Lagoa da Flecha	CPRM	46 km
Niquelândia	2,2%	Niquelândia	CEMADEN	<5 km
Nova Xavantina	0,9%	Nova Xavantina	INMET	<5 km
Novo Santo Antônio	1,3%	Vila Berrante	CPRM	60 km
Paraguaçu Paulista	3,4%	Lutércia	DAEE-SP	19 km
Paranatinga	3,4%	Passagem BR309	CPRM	17 km
Pedras de Maria Cruz	5,2%	Januária	INMET	14 km
Pedro Afonso	0,2%	Guaraí	CPRM	40 km
Pedro Leopoldo	1,3%	Ponte Raul Soares	CPRM	14 km
Peixe	2,5%	Peixe	CEMADEN	<5 km
Pontalina	1,9%	Morrinhos	CPRM	40 km
Ponte Firme	0,1%	João Pinheiro	INMET	41 km
Porangatu	1,2%	Entroncamento São Miguel	CPRM	34 km
Porto Nacional	2,4%	Fátima	CPRM	50 km
Porto Uerê	0,6%	Presidente Epitácio	DAEE-SP	20 km
Porto Velho	2%	Garcias	CPRM	28 km
Ribas do Rio Pardo	5,9%	Alegre	CPRM	31 km
Ribeiro Gonçalves	0,1%	Fazenda Tigre	CPRM	45 km

Rondonópolis	2,5%	Vale Rico	CPRM	46 km
Salitre	0,3%	Serra do Salitre	CPRM	12 km
Santa Juliana	0,4%	Perdizes	CPRM	24 km
Santa Rita de Cássia	1,2%	Mansidão	DNOS	58 km
São Carlos	5,1%	Vila Carmen	CPRM	<5 km
São Gonçalo	0,1%	Lagoa das Pedras	CPRM	11 km
São Romão	0,2%	São Romão	INMET	< 5km
Taguatinga	7,6%	Ponte Alta do Tocantins	CPRM	34 km
Tesouro	2,5%	Guiratinga	CPRM	36 km
Torixoreu	1,9%	Baliza	CEMADEN	<5 km
Unaí	2,9%	Unaí	INMET	< 5km
Várzea da Palma	0,9%	Pedra de Santana	CPRM	26 km
Vau do Balsamo	0,8%	Sidrolândia	CPRM	45 km
